# Modulating Single‐Atom Pt Coordination for Enhanced Low‐Temperature Ammonia Fuel Cell Electrocatalysis

**DOI:** 10.1002/adma.202508371

**Published:** 2025-07-26

**Authors:** Tong Wu, Xingyu Wang, Qin Yang, Bingqing Wang, Ruoou Yang, Shin‐An Chen, Lo‐Yueh Chang, Sibo Wang, Fuqiang Huang, Ziyun Wang, Yanwei Lum

**Affiliations:** ^1^ Department of Chemical and Biomolecular Engineering National University of Singapore Singapore 117585 Republic of Singapore; ^2^ Centre for Hydrogen Innovations National University of Singapore Singapore 117580 Republic of Singapore; ^3^ School of Chemical Sciences The University of Auckland Auckland New Zealand; ^4^ National Synchrotron Radiation Research Center Hsinchu 30076 Taiwan; ^5^ State Key Lab of Metal Matrix Composites School of Materials Science and Engineering Shanghai Jiao Tong University Shanghai 200240 China; ^6^ Institute of Materials Research and Engineering Agency for Science Technology and Research (A^*^STAR) 2 Fusionopolis Way, Innovis #08‐03 Singapore 138634 Republic of Singapore

**Keywords:** ammonia, electrocatalysis, fuel cell, single‐atom

## Abstract

Low‐temperature direct ammonia fuel cells (DAFCs) can be used for the on‐demand generation of clean electricity. However, such systems have low efficiency due to the kinetically sluggish ammonia oxidation reaction (AOR) and oxygen reduction reaction (ORR). Prior reports have largely focused on Pt‐based electrocatalysts, however, their high cost motivates the need for simultaneously increasing activity whilst reducing the metal loading. Here, the design of a bifunctional Pt single‐atom catalyst (SAC) is reported, with enhanced catalytic activities compared to commercial Pt/C for both reactions. Notably, by modulating the Pt SAC coordination, the optimal catalyst (Pt‐DG‐1) displayed a high AOR mass activity of 1.23 A mg_Pt_^−1^ and ORR mass activity of 7.98 A mg_Pt_^−1^. This is then integrated into a DAFC as both the cathode and anode, achieving a peak power density of 21.8 mW cm^−2^ and low Pt mass loading of only 0.034 mg cm^−2^. In situ shell‐isolated nanoparticle‐enhanced Raman spectroscopy (SHINERS) experiments on Pt‐DG‐1 indicate a lower ^*^OH coverage under ORR conditions and suppressed formation of poisoning species ^*^NO_x_ under AOR conditions as additional reasons for its enhanced bifunctional catalytic activity. Importantly, the study demonstrates how SACs can be rationally designed for DAFC electrocatalysis.

## Graphical abstract

A bifunctional Pt single‐atom catalyst (Pt‐DG‐1) is discovered with enhanced ammonia oxidation reaction and oxygen reduction reaction mass activities compared to commercial Pt/C. It is shown that modulating the coordination environment of Pt single atoms is crucial towards dictating the electrocatalytic activity. The bifunctional capability of Pt‐DG‐1 is demonstrated as both the cathode and anode in a direct ammonia fuel cell.

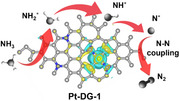

## 1 Introduction

Excessive consumption of fossil fuels for energy has brought about serious environmental concerns due to heavy CO_2_ emissions, which have caused increases in global temperatures.^[^ ^1^ ^]^ Hence, there is an urgent need to develop sustainable technologies that can decarbonize our global energy networks.^[^ ^2^ ^]^ In this context, green hydrogen is a promising option since it only generates water as a by‐product when consumed as fuel.^[^ ^3^ ^]^ However, a hydrogen economy cannot be realized until several technological challenges related to its safety, transportation, storage, and utilization are solved.^[^ ^4^ ^]^ As a result, there has been considerable interest in ammonia as a hydrogen carrier, with a high theoretical storage capacity of 17.6 wt.%.^[^ ^5^ ^]^

A direct ammonia fuel cell (DAFC) can be employed to release the chemical energy stored in ammonia.^[^ ^6^ ^]^ In such a system, the oxygen reduction reaction (ORR) occurs at the cathode and the ammonia oxidation reaction (AOR) occurs at the anode, which generates water and nitrogen gas as the only by‐products.^[^ ^7^ ^]^ Importantly, DAFCs allow for the on‐demand generation of electricity from ammonia. However, the efficiency of such systems is limited because of the sluggish kinetics of both the ORR and AOR.^[^ ^8^ ^]^ Currently, Pt remains the most promising and effective electrocatalyst for facilitating both reactions. For instance, Li et al. developed a catalyst consisting of ternary PtIrZn nanoparticles supported on CeO_2_, which was proposed to provide ^*^OH for promoting AOR.^[^ ^9^ ^]^ Recently, our group also designed a Ag_2_Pt_3_TiS_6_ catalyst featuring cooperative Pt and Ti sites with enhanced activity over commercial Pt/C for AOR.^[^ ^10^ ^]^ However, the scarcity and high cost of Pt are likely impediments to large‐scale deployment. Hence, improving the catalytic activity while substantially reducing the Pt metal loading content is of high importance.

For this purpose, transition metal single atom catalysts (SACs) are potentially attractive since these can, in principle, achieve 100% atom utilization.^[^ ^11^ ^]^ Such SACs can consist of metal atoms anchored and singly dispersed onto a conductive carbon‐based support.^[^ ^12^ ^]^ Due to their isolated nature, the catalytic properties of single atoms can differ considerably compared to the bulk metal.^[^ ^13^ ^]^ Furthermore, by modulating the coordination environments of the single metal atoms, their electronic configuration and hence catalytic activity can be tuned.^[^ ^14^ ^]^ Although SACs have been successfully developed for a broad range of reactions (e.g., CO_2_ reduction, H_2_ evolution), we surprisingly find, to the best of our knowledge, that there are no prior literature reports that have investigated SACs for facilitating the AOR.^[^ ^15^ ^]^

Motivated by this, we herein designed and synthesized a SAC consisting of Pt single atoms anchored onto a defective nitrogen‐doped graphene support for AOR. We found that modulating the vacancy defects of the support allows for tuning of the coordination environment and hence catalytic activity of the Pt SACs. Notably, we find that the optimal SAC (Pt‐DG‐1) exhibits enhanced electrocatalytic performance for both AOR and ORR as compared to commercial Pt/C. Through comprehensive density functional theory (DFT) calculations, we study the impact of various coordination environments on how Pt SACs bind reaction intermediates and the resulting reaction pathway energetics. In situ shell‐isolated nanoparticle‐enhanced Raman spectroscopy (SHINERS) experiments on Pt‐DG‐1 reveal that the decreased propensity for the formation of ^*^NO_x_ poisoning species as an additional reason for its enhanced AOR activity. Finally, we integrated Pt‐DG‐1 as both the cathode and anode of a DAFC and successfully achieved a high peak power density of 21.8 mW cm^−2^ with low Pt mass loading of 0.034 mg cm^−2^, outperforming most prior literature reports.

## 2 Results and Discussion

### 2.1 Catalyst Materials Characterization

**Figure 1:**
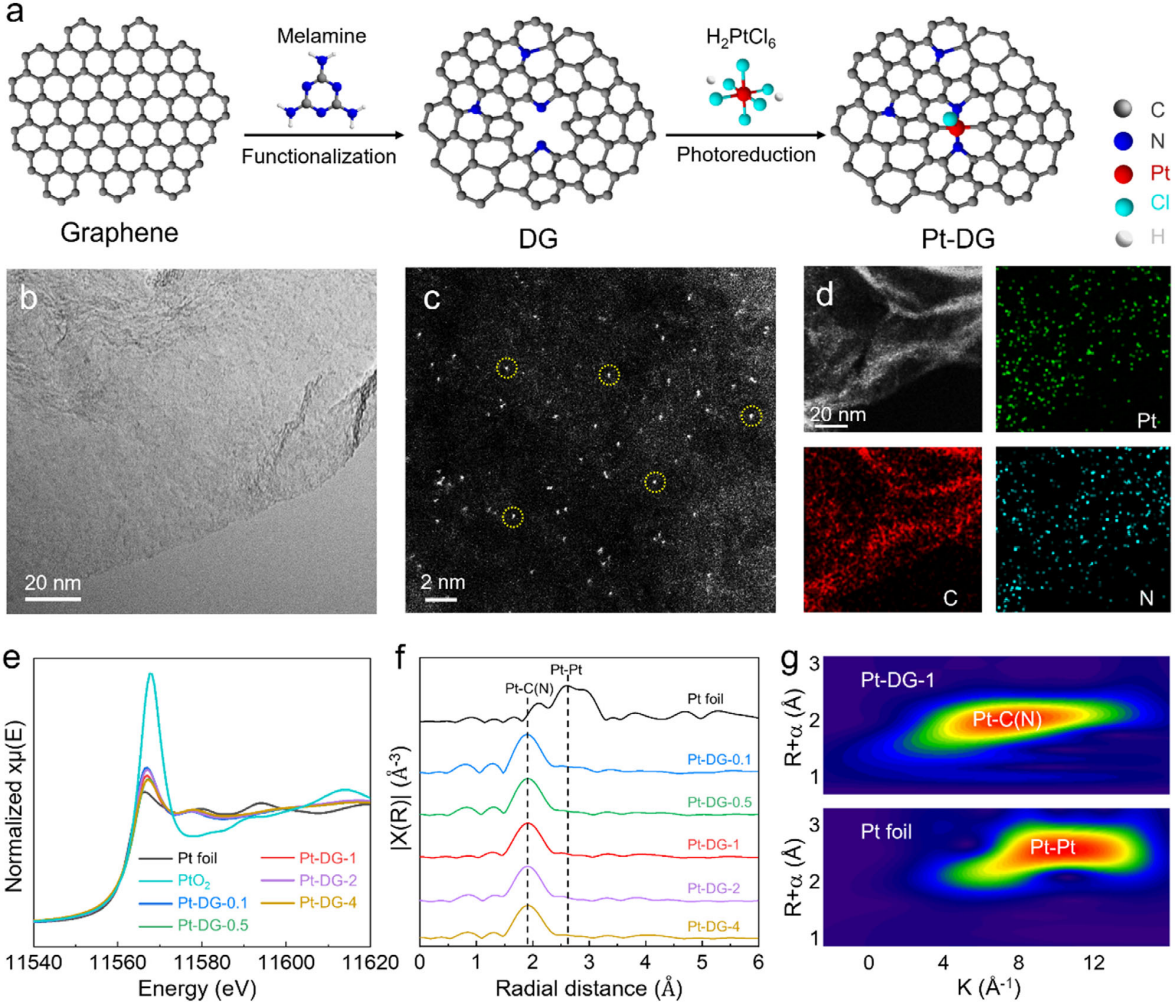
Structural analysis and physical characterization of Pt‐DG‐x. a) Schematic illustration of the preparation of Pt‐DG. b) TEM, c) aberration‐corrected high‐angle annular dark‐field STEM, d) EDS mapping images of Pt‐DG‐1, with Pt in green, C in red, and N in blue. e) Pt L‐edge XANES spectra of Pt‐DG‐x, PtO_2_, and Pt foil. f) R‐space Pt L‐edge EXAFS spectra of Pt‐DG‐x and Pt foil. g) Wavelet transforms for the k2‐weighted Pt L‐edge EXAFS of Pt‐DG‐1 and Pt foil.

Pt SACs were synthesized according to a previous literature method by Yang et al.^[^ ^16^ ^]^ As shown in **Figure** **1**a, melamine was mixed with pristine graphene and heated at 700 °C under an inert environment to form nitrogen‐doped graphene. Further heating at 1150 °C results in partial nitrogen loss, leaving behind a defective graphene support. The vacancy defect density can be tuned by varying the amount of melamine added. Finally, Pt single atoms were anchored onto the defective graphene support by photoreduction of H_2_PtCl_6_ using a Xenon lamp (see Methods section for full synthesis procedure). In this work, five different Pt SACs of varying defect density were synthesized, which are termed as Pt‐DG‐x where “x” refers to the melamine ratio.

X‐ray diffraction (XRD) patterns of Pt‐DG‐0.1, Pt‐DG‐0.5, Pt‐DG‐1, Pt‐DG‐2, and Pt‐DG‐4 indicate the absence of any metallic Pt phases, with only two broad overlapping peaks assigned to graphitic carbon for all samples (Figure S1, Supporting Information). Inductively coupled plasma‐optical emission spectroscopy (ICP‐OES) analysis was then used to assess the Pt composition (Figure S2, Supporting Information). From this, the Pt contents were all found to be similar, with values of 2.02%, 1.65%, 1.67%, 1.86%, and 2.23 wt.% for Pt‐DG‐0.1, Pt‐DG‐0.5, Pt‐DG‐1, Pt‐DG‐2, and Pt‐DG‐4, respectively.

To investigate the degree of graphitization, we performed Raman spectroscopy (Figure S3, Supporting Information) to examine the intensity ratio of defect‐related D band at 1350 cm^−1^ and graphitic carbon‐related G band at 1600 cm^−1^ (*I*_D_ */I*_G_). The *I*_D_ */I*_G_ values of Pt‐DG‐0.1, Pt‐DG‐0.5, Pt‐DG‐1, Pt‐DG‐2, and Pt‐DG‐4 were found to be 1.09, 1.10, 1.13, 1.21, and 1.18, respectively. We note that the defect density gradually increases from Pt‐DG‐0.1 to Pt‐DG‐1, which is due to a higher melamine loading used in the synthesis procedure. However, the reverse trend was observed for Pt‐DG‐4 with a lower than expected *I*_D_ */I*_G_ value, which we speculate to be due to defect reconstruction.

Transmission electron microscopy (TEM) of Pt‐DG‐1 shows the layered structure of the graphene support without any observable Pt nanoparticles or clusters (Figure 1b and Figure S4, Supporting Information). To observe the isolated Pt single atoms, aberration‐corrected high‐angle annular dark‐field scanning TEM (HAADF‐STEM) was performed. From the image (Figure 1c), the Pt single atoms appeared as bright dots dispersed across the graphene support. TEM and HAADF‐STEM characterization of Pt‐DG‐0.1, Pt‐DG‐0.5, Pt‐DG‐2, and Pt‐DG‐4 were also carried out, with similar observations and results (Figures S5–S8, Supporting Information). In addition, energy dispersive X‐ray spectroscopy (EDX) shows the homogeneous spatial distributions of platinum, carbon, and nitrogen on Pt‐DG‐1 (Figure 1d).

To investigate the chemical state of the Pt‐DG‐x samples, X‐ray photoelectron spectroscopy (XPS) was carried out (Figures S9–S12, Supporting Information). As shown in Pt 4*f* XPS spectra of Pt‐DG‐x (Figure S10, Supporting Information), two peaks at 72.3 and 75.5 eV correspond to Pt^2+^ 4*f*_7/2_ and Pt^2+^ 4*f*_5/2_, respectively. Another two peaks at 73.6 and 77.5 eV correspond to Pt^4+^ 4*f*_7/2_ and Pt^4+^4*f*_5/2_, respectively. From this, we determined that 86.3% of Pt‐DG‐1 are Pt^2+^, with the remainder being Pt^4+^. As for the other Pt‐DG‐x, the Pt^2+^ ratio was determined to be 79.6% for Pt‐DG‐0.1, 81.3% for Pt‐DG‐0.5, 78.2% for Pt‐DG‐2 and 70.2% for Pt‐DG‐4.

The N 1*s* spectra of Pt‐DG‐x in Figure S11 (Supporting Information) shows the presence of pyridinic N (398.1 eV), pyrrolic N (399.9 eV), graphiti N (401.6 eV), and oxidized N (404.2 eV). To examine the N coordination environment with Pt atoms, the spectral deconvolution of each type is obtained (Table S1, Supporting Information). The pyrrole N of Pt‐DG‐1 was 29.2%, higher than that of Pt‐DG‐0.1 (20.5%), Pt‐DG‐0.5 (24.1%), Pt‐DG‐2 (19.3%), and Pt‐DG‐4 (13.1%). For comparison, the pyridine N of Pt‐DG‐1 (19.4%) is the lowest among all Pt SACs. Hence, these results indicate that the N coordination environment of Pt atoms of Pt‐DG‐1 is different from the other Pt‐DG‐x samples. In the Cl 2*p* spectra (Figure S12, Supporting Information), two peaks at 197.7 and 199.5 eV corresponding to 2*p*_3/2_ and 2*p*_1/2_ were observed, indicating the presence of Cl–Pt bonding.

To further understand the local coordination structure of Pt atoms, we performed X‐ray absorption spectroscopy (XAS) on the samples. Pt L_3_‐edge X‐ray near‐edge structure (XANES) spectra of the Pt‐DG‐x, PtO_2_, and Pt foil are shown in Figure 1e. The whiteline intensities of Pt‐DG‐x are between those of Pt foil and PtO_2_, indicating that Pt has a positive oxidation state and is consistent with the XPS results. Fourier‐transformed (FT) k2‐weighted extended X‐ray absorption fine structure (EXAFS) oscillations of Pt‐DG‐x and Pt foil are shown in Figure 1f. First, the Pt foil exhibits the peak at 2.60 Å, which is ascribed to the Pt‐Pt bond. On the other hand, we did not observe any peaks that could be attributed to Pt‐Pt bonds in the Pt‐DG‐x samples, which provides support for their single‐atom nature. The peak at approximately 1.85 Å in Pt‐DG‐x was attributed to Pt–C(N) and Pt–Cl coordination.

As shown in Figure S13 (Supporting Information), the coordination number (CN) of Pt‐DG‐x are obtained by EXAFS fitting (Pt‐C(N) and Pt‐Cl shells). The oscillation curves of the Pt L_3_‐edge for Pt‐DG‐x in the K range of 0.0–12.0 Å^−1^ are also shown in Figure S14 (Supporting Information). To further investigate the corresponding coordination environments of Pt, wavelet transform (WT) analysis was performed (Figure 1g and Figure S15, Supporting Information). The Pt‐DG‐x samples all display an intensity maximum at ≈1.85 Å^−1^, which is ascribed to Pt‐C(N) coordination. The best fitting values of structural parameters are listed in Table S2 (Supporting Information), which all contain coordination with both C(N) and Cl. From the results, we find that the coordination number of the Pt atom decreases with increasing melamine content. For instance, the Pt atom in Pt‐DG‐0.1, Pt‐DG‐0.5, and Pt‐DG‐1 all have a coordination number of ≈5, consisting of ≈4 coordinating C(N) atoms and 1 Cl atom. This coordination number then decreases to ≈4 with Pt‐DG‐2 and Pt‐DG‐4, with ≈3 coordinating C(N) atoms and 1 Cl atom.

Importantly, these results indicate that varying the melamine content can indeed regulate the Pt SAC coordination environment and hence can serve as a platform to study its impact on the observed AOR and ORR activity.

### 2.2 Electrocatalytic Activity Evaluation

**Figure 2:**
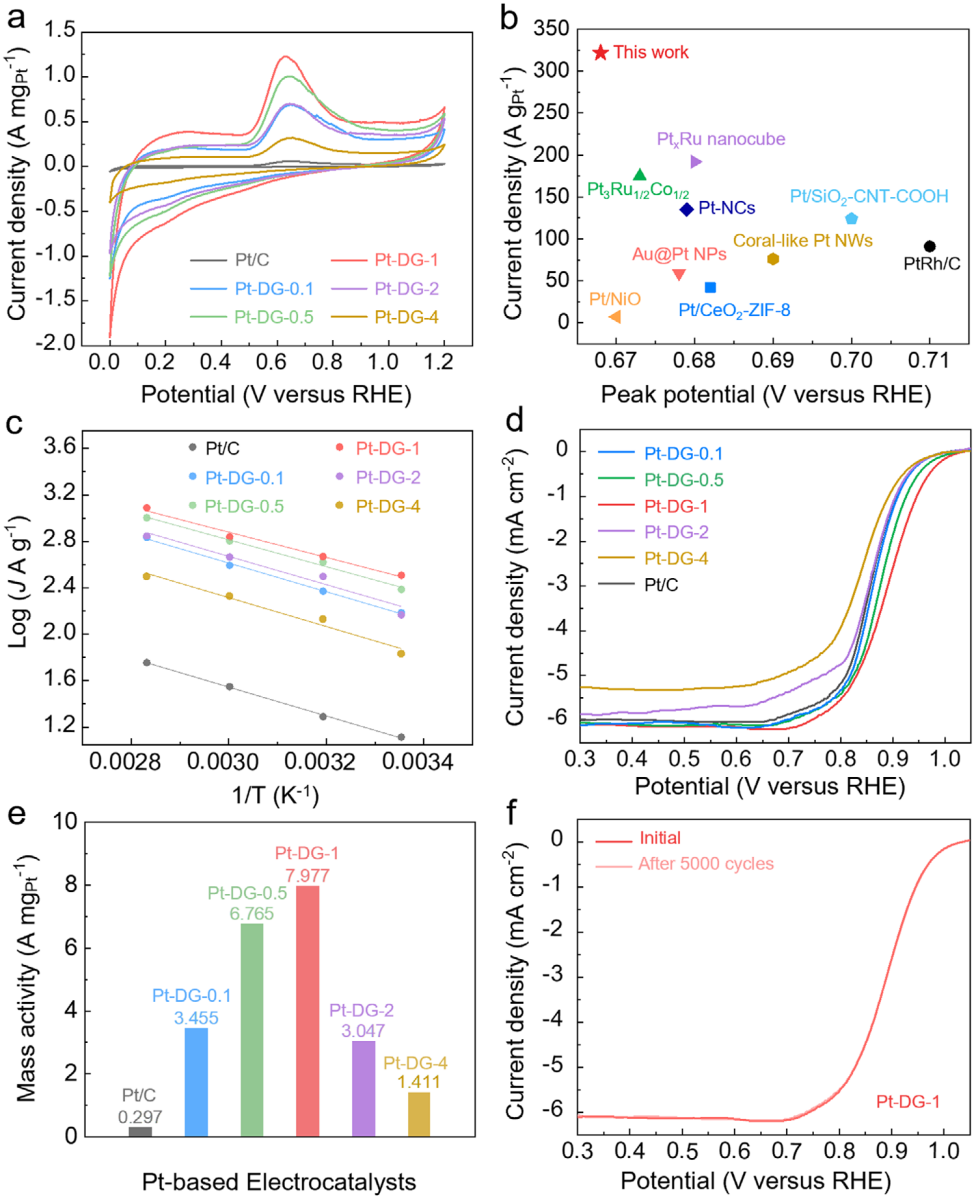
Electrocatalytic performance characterizations. a) AOR CV curves of Pt‐DG‐x and Pt/C obtained using 0.1 m NH_4_OH in 1.0 m KOH. b) Comparison of the current density and peak potential of the Pt‐DG‐1 with other reported Pt‐based catalysts at room temperature. c) Arrhenius plots for NH_3_ oxidation on Pt‐DG‐x and Pt/C at 0.7 V versus RHE. d) ORR polarization curves of Pt‐DG‐x and Pt/C. ORR measurements were performed in O_2_ saturated 0.1 m KOH electrolyte using an RDE at 1600 rpm. e) Mass activity of Pt‐DG‐x and Pt/C at 0.85 V versus RHE. f) ORR polarization curves before and after 5000 cycles of Pt‐DG‐1 in 0.1 m KOH.

The AOR activity of Pt‐DG‐x was assessed using cyclic voltammetry (CV) in 1.0 M KOH with 0.1 m NH_4_OH electrolyte between 0.0 and 1.2 V versus RHE. For comparison, similar CV experiments were also performed with commercial Pt/C. From the results (**Figures** **2**a and S16, Supporting Information), the onset potential of Pt‐DG‐x is ≈0.47–0.50 V versus RHE (Table S3, Supporting Information), which is lower than Pt/C (0.522 V). In addition, the AOR peak mass activity of Pt‐DG‐1 was 1.23 A mg_Pt_^−1^, which is much higher than that of Pt‐DG‐0.1 (0.70 A mg_Pt_^−1^), Pt‐DG‐0.5 (1.01 A mg_Pt_^−1^), Pt‐DG‐2 (0.69 A mg_Pt_^−1^), Pt‐DG‐4 (0.38 A mg_Pt_^−1^) and Pt/C (0.057 A mg_Pt_^−1^). Hence, it appears that a higher defect density of the carbon support increases the AOR activity of the Pt SAC from Pt‐DG‐0.1 to Pt‐DG‐1. However, a higher defect density with Pt‐DG‐2 and Pt‐DG‐4 actually leads to a lowered AOR activity. This volcanic relationship between AOR activity and defect density suggests that there is an optimal coordination environment for the Pt SAC where intermediate adsorption energies are most ideal. Furthermore, in comparison to prior literature reported Pt‐based electrocatalysts, Pt‐DG‐1 demonstrates higher AOR mass activity (Figure 2b and Tables S4,S5, Supporting Information).

To examine whether any agglomeration occurs, we performed HAADF‐STEM characterization of the catalyst after the experiment. As shown in Figure S17 (Supporting Information), post‐reaction Pt‐DG‐1 still exhibits Pt single atoms, which appear as bright dots dispersed across the graphene support. To investigate the effect of varying the carbon support material, we loaded Pt single atoms onto defective carbon nanotubes (DCNT) using a similar method. In Figure S18 (Supporting Information), the AOR peak mass activities of Pt‐DCNT‐0.1, Pt‐DCNT‐1 and Pt‐DCNT‐4 were 0.37, 0.27, and 0.23 A mg_Pt_^−1^, respectively, which are much lower than that of Pt‐DG‐1 (1.23 A mg_Pt_^−1^). This indicates that different carbon materials will have a considerable impact on the coordination environment of the Pt single atoms, which then influence the observed AOR activity.

These measurements were then repeated at a range of higher operating temperatures, with the corresponding CV curves of Pt‐DG‐x and Pt/C shown in Figure S19 (Supporting Information). Consistent with expectations, we found that the AOR peak mass activity increases with a higher temperature. For instance, the AOR peak mass activity of Pt‐DG‐1 increases from 0.68 to 1.23 A mg_Pt_^−1^ at temperatures of 25 and 80 °C, respectively. From these results, the Arrhenius plots of Pt‐DG‐x and Pt/C for AOR at 0.7 V versus RHE were derived (Figure 2c). Based on this, the activation energy of Pt‐DG‐1 was calculated to be 8.29 kJ mol^−1^, which is lower than that of Pt‐DG‐0.1 (9.89 kJ mol^−1^), Pt‐DG‐0.5 (9.04 kJ mol^−1^), Pt‐DG‐2 (10.11 kJ mol^−1^), Pt‐DG‐4 (10.32 kJ mol^−1^) and Pt/C (10.61 kJ mol^−1^). This suggests that the activation energy barrier in the rate‐determining step for AOR is lowest for the Pt‐DG‐1 catalyst.

Next, we sought to evaluate the ORR activity of Pt‐DG‐x by performing linear sweep voltammetry (LSV) using a rotating disk electrode (RDE) in O_2_‐saturated 0.1 m KOH electrolyte at room temperature. As shown in Figure 2d and Figure S20 (Supporting Information), the half‐wave potential (E_1/2_) of Pt‐DG‐1 is 0.887 V versus RHE, which is higher than that of Pt‐DG‐0.1 (0.867 V), Pt‐DG‐0.5 (0.871 V), Pt‐DG‐2 (0.868 V), Pt‐DG‐4 (0.844 V), Pt/C (0.858 V), and bare DG‐1 (0.778 V). In Figure 2e, the mass activity of Pt‐DG‐1 catalyst is 7.977 A mg_Pt_^−1^, which is higher than that of Pt‐DG‐0.1 (3.455 A mg_Pt_^−1^), Pt‐DG‐0.5 (6.765 A mg_Pt_^−1^), Pt‐DG‐2 (3.047 A mg_Pt_^−1^), Pt‐DG‐4 (1.411 A mg_Pt_^−1^) and Pt/C (0.297 A mg_Pt_^−1^).

It is intriguing that Pt‐DG‐1 was found to have the highest activity for both ORR and AOR. Similarly, in our previous work, the AOR catalyst (Ag_2_Pt_3_TiS_6_) that we developed was also found to possess good ORR capability. Based on prior literature, theoretical calculations highlighted a potential role of ^*^OH in stabilizing the crucial ^*^NH intermediate via hydrogen bonding. At the same time, the formation and coverage of ^*^OH are also known to have a considerable impact on ORR kinetics. Based on these observations, we speculate that AOR and ORR could have shared activity descriptors, which motivates the need for future studies to further understand this.

Next, a stability test consisting of 5000 CV cycles was conducted to investigate the robustness of Pt‐DG‐1. From the results (Figure 2f and Figure S21, Supporting Information), the E_1/2_ value was found to be negatively shifted by only 1 mV after 5000 CV cycles, indicating good catalyst stability. In comparison, similar tests conducted with the commercial Pt/C catalyst showed a larger shift of 8 mV.

**Figure 3:**
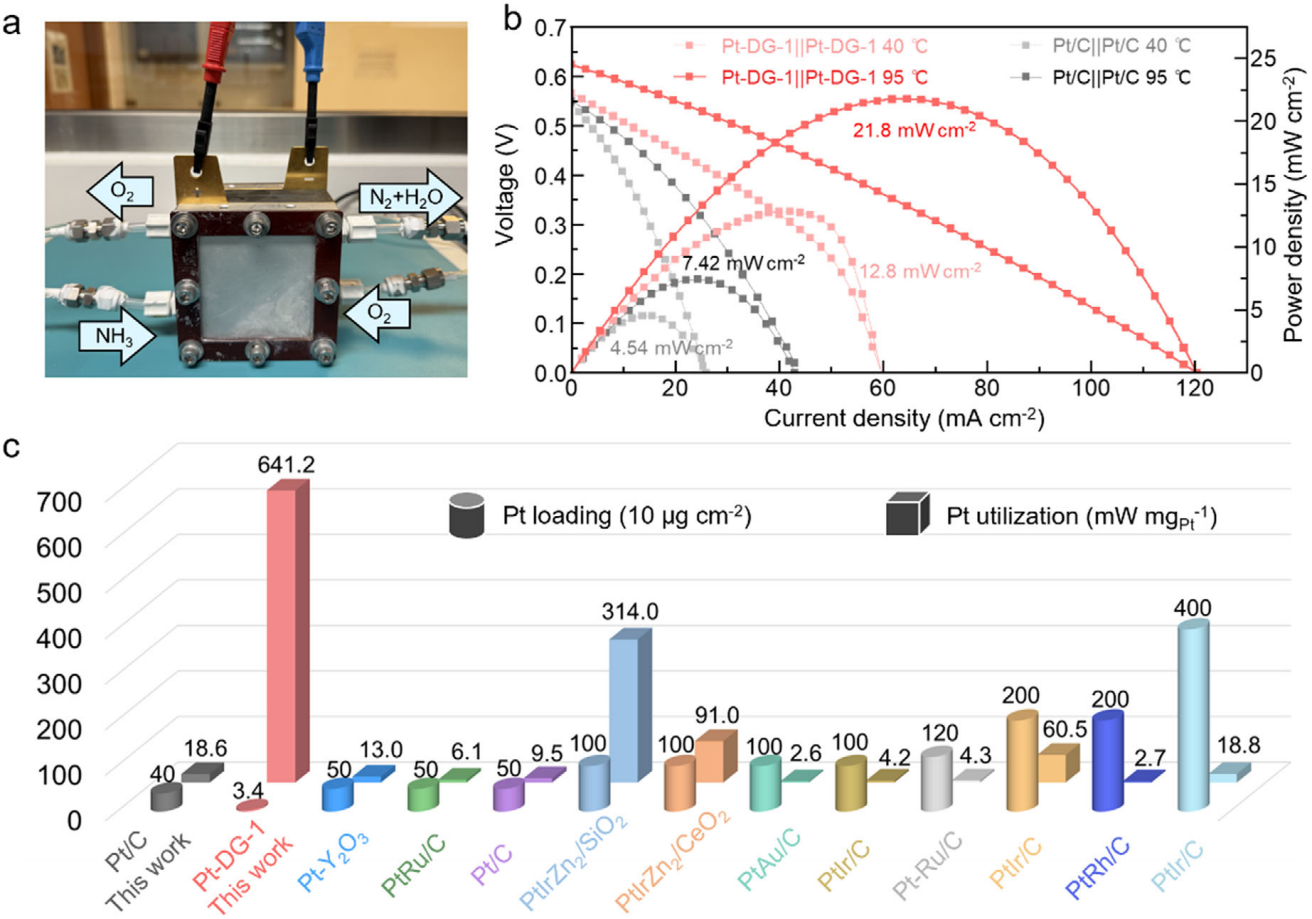
DAFC performance with Pt‐DG‐1. a) Photograph of the 5 cm^2^ DAFC used in our work. b) Polarization and power density curves of the DAFC at 40 and 95 °C using Pt‐DG‐1 and Pt/C with 5 m NH_4_OH in 1 m KOH. c) Comparison of the Pt utilization of Pt‐DG‐1||Pt‐DG‐1 with other reported Pt‐based electrocatalysts.

We then employed Pt‐DG‐x as both the cathode (ORR) and anode (AOR) in a DAFC (**Figure** **3**a). The polarization and power density curves of Pt‐DG‐1 and Pt/C in 1.0 mol L^−1^ KOH with 5.0 mol L^−1^ NH_4_OH at 40 and 95 °C are shown in Figure 3b. The open‐circuit voltage (OCV) of Pt‐DG‐1 are 0.566 and 0.624 V at 40 and 95 °C, respectively. These are higher than Pt/C with values of 0.544 and 0.551 V at 40 and 95 °C, respectively. The OCVs for all of Pt‐DG‐x are shown in Figure S22 (Supporting Information) for 40 °C and Figure S23 (Supporting Information) for 95 °C. Notably, the DAFC with Pt‐DG‐1 has the highest OCV, which is due to its highest AOR and ORR kinetics.

In addition, the peak power density of the DAFC with Pt‐DG‐1 is 12.8 mW cm^−2^ at 40 °C with a low Pt mass loading of 0.034 mg_Pt_ cm^−2^. These values are higher than the DAFC with Pt/C (4.54 mW cm^−2^, 0.4 mg_Pt_ cm^−2^). When the DAFC with Pt‐DG‐1 was measured at 95 °C, the peak power density increased to 21.8 mW cm^−2^, which is also higher than the DAFC with Pt/C (7.42 mW cm^−2^) and the DAFC with other Pt‐DG‐x. Notably, we also find that the Pt utilization of the DAFC with Pt‐DG‐1 is 641.2 mW mg_Pt_^−1^, which outperforms that of many previously reported DAFCs (Figure 3c and Tables S6,S7, Supporting Information).

### 2.3 In Situ Experiments with SHINERS

**Figure 4:**
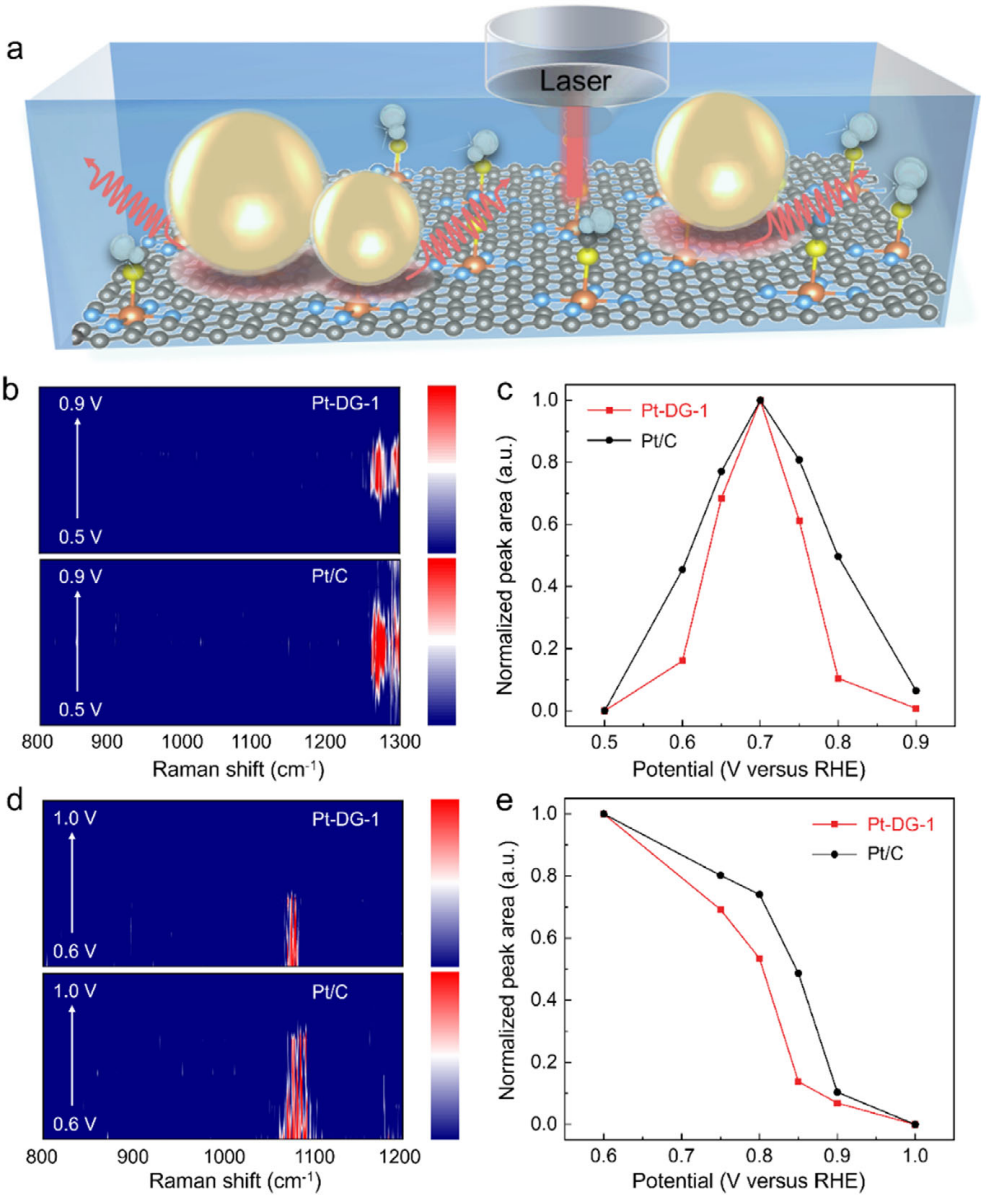
In situ SHINERS for detecting surface adsorbates. a) Schematic of In situ SHINERS experiments which utilize SiO_2_‐coated Au nanoparticles to enhance the Raman signal. b) Potential‐dependent Raman contour maps of Pt‐DG‐1 and Pt/C obtained during AOR at the potential from 0.5 to 0.9 V versus RHE. c) Normalized peak area of the band attributed to ^*^NO_x_ on Pt‐DG‐1 and Pt/C during AOR at the potential from 0.5 to 0.9 V versus RHE. d) Potential‐dependent Raman contour maps of Pt‐DG‐1 and Pt/C obtained during ORR in the potential range from 1.0 to 0.6 V versus RHE. e) Normalized peak area of ^*^OH on Pt‐DG‐1 and Pt/C obtained during ORR in the potential range from 1.0 to 0.6 V versus RHE.

To detect surface adsorbates on Pt‐DG‐1 and Pt/C under AOR conditions, In situ shell‐isolated nanoparticle‐enhanced Raman spectroscopy (SHINERS) was performed in the range of 0.5 to 0.9 V versus RHE. This technique^[^ ^17^ ^]^ uses SiO_2_‐coated Au nanoparticles added on the catalyst surface to enhance the Raman signal (**Figure** **4**a). As shown in Figure 4b and Figure S24 (Supporting Information), a peak at 1250–1300 cm^−1^ was observed, which can be assigned to adsorbed ^*^NO_x_ species on the catalyst surface.^[^ ^18^ ^]^ These have been previously proposed to be a poisoning species due to their tendency to form strong bonds to active sites.^[^ ^19^ ^]^ To provide a comparison with Pt/C, we normalized the area of this peak for both catalysts and plotted this as a function of potential in Figure 4c. The highest intensity occurs at 0.7 V versus RHE for both Pt‐DG‐1 and Pt/C, which coincides with the peak AOR current in CV experiments. Notably, we observe that the normalized peak area for Pt‐DG‐1 grows slower and then decays faster as compared to Pt/C when the applied potential is gradually increased from 0.5 to 0.9 V versus RHE. This suggests that Pt‐DG‐1 accumulates a lower coverage of ^*^NO_x_ and hence is less affected by poisoning as compared to Pt/C. Hence, this provides another reason for the higher AOR activity of Pt‐DG‐1 as compared to Pt/C.

Similar experiments with Pt‐DG‐1 and Pt/C were also performed for ORR as shown in Figure 4d and Figure S25 (Supporting Information). This was carried out in the applied potential range of 1.0 to 0.6 V versus RHE, where a peak at ≈1060 cm^−1^ was observed corresponding to ^*^OH on the catalyst surface.^[^ ^20^ ^]^ Similarly, this peak area was also normalized in Figure 4e to facilitate comparison between Pt‐DG‐1 and Pt/C. The accumulation of ^*^OH on Pt‐DG‐1 is shifted in a cathodic direction as compared to Pt/C, which demonstrates a lower coverage of ^*^OH on Pt‐DG‐1 during ORR. Notably, a lower ^*^OH coverage has been previously postulated to be beneficial towards ORR kinetics,^[^ ^21^ ^]^ which was also supported by our DFT calculations (Figures S26–S29, Supporting Information). Hence, this observation also provides another explanation for the higher ORR activity of Pt‐DG‐1 as compared to Pt/C.

### 2.4 Density Functional Theory Calculations

We next employed density functional theory (DFT) calculations to understand how varying coordination environments of Pt SACs can influence the AOR activity. For this, we built computational models of 8 different single‐atom Pt active site configurations embedded in graphene supports as shown in Figures S30–S38 (Supporting Information). These structures differ in their coordination environments, with Pt‐4N2Cl‐1 through Pt‐4N2Cl‐5 showing four‐coordinated platinum centers, while Pt‐5N2Cl‐1, Pt‐5N2Cl‐2, and Pt‐5N3Cl‐3 display five‐coordinated platinum centers with different arrangements of C, N, and Cl atoms around the central Pt atom. To understand how different active site configurations affect the reaction pathway, we conducted first‐principles calculations to obtain Gibbs free energy profiles (details in the Methods section). Here, we compared the eight different SAC configurations with Pt(111) as a reference catalyst surface for comparison.

**Figure 5:**
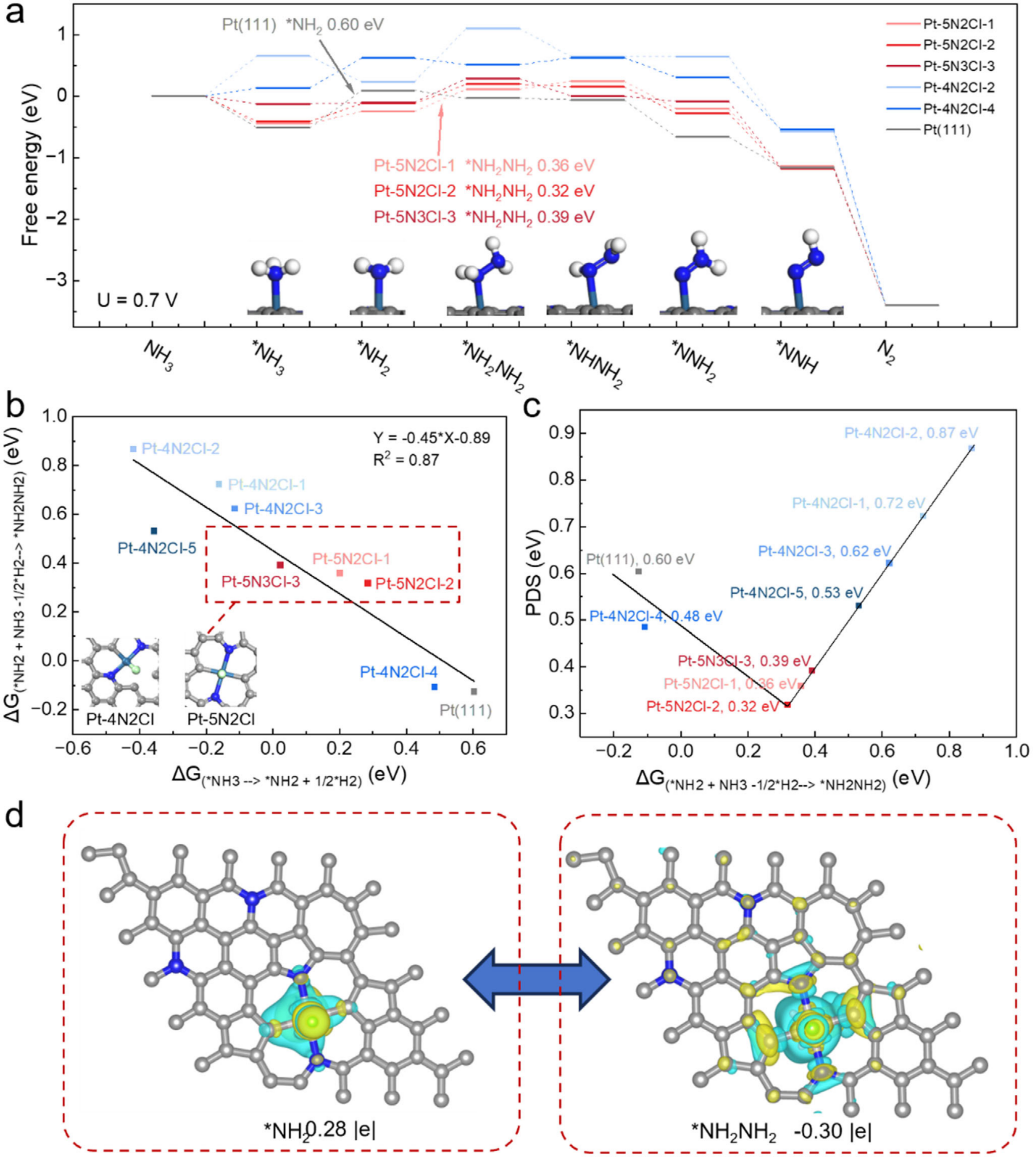
Computational investigation of the reaction mechanism. a) Free energy diagram showing the pathway of NH_3_ oxidation to N_2_ involving the intermediates ^*^NH_3_, ^*^NH_2_, ^*^NH_2_NH_2_, ^*^NHNH_2_, ^*^NNH_2_ and ^*^NNH at 0.7V versus RHE. b) The relationship between the free energy change (∆G) of ^*^NH_3_ → ^*^NH_2_ + 1/2H_2_ and the free energy change (∆G) of ^*^NH_2_+NH_3_→ ^*^NH_2_NH_2_ + 1/2H_2_ for various sites. Examples of four‐coordinated and five‐coordinated catalyst structures are shown in the bottom left corner. c) Volcano plot showing the relationship between the free energy of the potential‐determining step and the free energy change (∆G) of ^*^NH_2_ →^*^NH_2_NH_2_. d) Differential charge density maps of ^*^NH_2_ and ^*^NH_2_NH_2_ adsorbed on the Pt‐5N2Cl‐2, illustrating electron transfer between the substrate and adsorbates.

**Figures** **5**a and S39 (Supporting Information) demonstrate the catalyst coordination structure's significant impact on reaction activity (convergence tests shown in Figure S40, Supporting Information). The figure presents the complete reaction pathway for ammonia (NH_3_) oxidation to nitrogen (N_2_), which proceeds through the intermediates: ^*^NH_3_, ^*^NH_2_, ^*^NH_2_NH_2_, ^*^NHNH, ^*^NNH_2_, ^*^NNH and N_2_. First, the five‐coordinated sites (Pt‐5N2Cl‐1, Pt‐5N2Cl‐2, and Pt‐5N2Cl‐3) show marked energetic advantages over the four‐coordinated sites, as evidenced by their lower potential determining step (PDS) energies.^[^ ^22^ ^]^ Specifically, the energies of PDS for five‐coordinated structures range from 0.32 to 0.39 eV, while those for the four‐coordinated structures are observed to be from 0.48 to 0.87 eV.

Notably, while these structures follow the same reaction pathway, they exhibit different PDS in the overall mechanism. For the Pt(111), the ^*^NH_3_ → ^*^NH_2_ step is the PDS with a free energy change of 0.60 eV, whereas for all five‐coordinated structures, the ^*^NH_2_ → ^*^NH_2_NH_2_ step becomes the PDS. Overall, the reaction on all five‐coordinated SACs exhibits lower free energy than that on Pt(111). This indicates that modulating the coordination environment of the Pt single‐atom can indeed influence the AOR activity.

Importantly, these results provide an explanation for our experimental trends where we found there was an optimal coordination environment (Pt‐DG‐1) that displayed the highest AOR kinetics. Specifically, Pt‐DG‐1 was fitted with a coordination number of ≈4.9 (Table S2, Supporting Information), which is close to our predicted optimal value of 5 from DFT calculations. Although Pt‐DG‐0.1 and Pt‐DG‐0.5 also have fitted coordination numbers close to 5, it is likely that these have slight differences in the arrangements of N, C, and Cl, which result in less optimal AOR kinetics. As for Pt‐DG‐2 and Pt‐DG‐4, their fitted coordination numbers are ≈4 and hence display lower AOR kinetics as predicted by our DFT calculations.

Through analysis of reaction activity on all SACs, a significant correlation was also discovered between the free energy changes of two key steps: ^*^NH_3_ → ^*^NH_2_ + 1/2H_2_ and ^*^NH_2_+NH_3_→ ^*^NH_2_NH_2_ + 1/2H_2_. As shown in Figure 5b, these steps exhibit a clear negative correlation (R^2^ = 0.87). Depending on the specific catalyst, either of these steps can become PDS. This indicates that when the free energy change of ^*^NH_2_ formation is too high, this step becomes the PDS. Conversely, the ^*^NH_2_ coupling step to form ^*^NH_2_NH_2_ becomes the PDS. This therefore predicts that the ideal catalyst should have balanced free energy changes for these two key steps to minimize the overpotential associated with PDS. As shown in Figure 5b, all the five‐coordinated active site configurations (highlighted regions) meet this important condition.

We also found a “U‐shaped” relationship (Figure 5c) between the free energy change of the PDS and the free energy change of the ^*^NH_2_NH_2_ coupling step. When the free energy change (ΔG) of ^*^NH_2_ coupling to form ^*^NH_2_NH_2_ is 0.29 eV on Pt‐5N2Cl‐2, the PDS energy reaches its minimum value (0.32 eV). Near this energetic optimum, other five‐coordinated environments such as Pt‐5N2Cl‐1 and Pt‐5N2Cl‐3 also exhibit excellent catalytic activity. Deviations from this optimal free energy change of coupling steps in either direction result in increased PDS energy values, consequently reducing catalytic performance.

To gain deeper insights into the optimal Pt‐5N2Cl‐2 coordination environment, we performed differential charge density analysis (Figure 5d). The results show electron transfer of 0.28|e| between ^*^NH_2_ and Pt‐5N2Cl‐2, while the ^*^NH_2_NH_2_ coupling step involves an electron transfer of 0.30|e|. The stronger adsorption interaction between ^*^NH_2_NH_2_ and Pt‐5N2Cl‐2 increases the free energy change required for its further transformation, thus making the ^*^NH_2_NH_2_ coupling step the PDS.

## 3 Conclusion

In this work, our objective was to develop single‐atom Pt catalysts for ammonia fuel cell electrocatalysis. For this, we first used a high‐temperature pyrolysis method to create defective nitrogen‐doped carbon supports. Pt atoms were then singly dispersed onto the support and coordinated to C, N, and Cl atoms using a photoreduction method with H_2_PtCl_6_. Notably, by adjusting the pyrolysis temperature, we were able to develop SACs with varying coordination environments. In particular, Pt‐DG‐1 with 1.67 wt% Pt loading showed higher performance compared to commercial Pt/C for both AOR and ORR. Specifically, it displayed a high AOR mass activity of 1.23 A mg_Pt_^−1^ and ORR mass activity of 7.98 A mg_Pt_^−1^. SHINERS experiments show a reduced coverage of ^*^NO_x_ species on Pt‐DG‐1 during the AOR, which indicates an increased resistance towards catalyst poisoning compared to Pt/C. Density functional theory calculations identified that balancing the free energy change of the ^*^NH_2_ formation step and the ^*^NH_2_ coupling step to be key towards optimizing AOR activity, which can best be achieved using five‐coordinated Pt environments. Finally, we assembled Pt‐DG‐1 as both the cathode and anode in a DAFC and achieved a high peak power density of 21.8 mW cm^−2^. Notably, the DAFC attained a high Pt utilization of 641.2 mW mg_Pt_^−1^ and low Pt mass loading of 0.034 mg cm^−2^, which outperforms most prior literature reports.

## 4 Experimental Section

### Materials

All chemicals were used as purchased without further purification. Potassium hydroxide (ACS reagent, ≥85%, pellets), ethanol (ACS reagent, ≥99.5%), platinum black (powder, ≥99.95% trace metals basis), melamine (analytical standard,

≥99.0%), chloroplatinic acid hexahydrate (ACS reagent, ≥37.50% Pt basis), ammonium hydroxide solution (ACS reagent, 28.0‐30.0% NH_3_ basis), and isopropyl alcohol (ACS reagent, ≥99.5%) were purchased from Sigma–Aldrich. Aemion (TM) reinforced anion exchange membrane, (AF3‐HWK9‐75‐X 75 µm) and carbon paper (AvCarb, GDs3250) were purchased from CTECH Global Pte. Ltd. Graphitized carbon (nano powder, graphitized, <500 nm particle size, ≥99.95% trace metals basis) and Nafion perfluorinated resin solution (5.0–5.4%) were purchased from Aladdin. Graphene (Flake diameter 0.5–5µm, thickness ≈0.8nm, single‐layer rate 80%) was purchased from Nanjing Xianfeng Nano.

### The Synthesis of D‐Graphene

DG was obtained from pristine graphene mixed with melamine using the thermal annealing method. In a typical method, the graphene was mixed with melamine (mass ratio are 1:0.1, 1:0.5, 1:1, 1:2, 1:4, respectively) annealed at 700 °C for 2 h, and then annealed at 1150 °C for 2 h with a ramp rate of 5 °C under an atmosphere of nitrogen. The as prepared samples were denoted as DG‐0.1, DG‐0.5, DG‐1, DG‐2, DG‐4.

### The Synthesis of Pt@DG

In a facile method, DG (30 mg) was added in 20 mL of ethanol in a 50 mL beaker, the beaker was placed into an ultrasonic cleaning machine ultrasounded at room temperature for 0.5 h. Then Chloroplatinic acid hexahydrate (0.9 mg) was added into the turbid liquid and lighted by the xenon lamp for 10 min, after washing with deionized water for three times, the prepared sample was dried, and Pt‐DG‐x was finally obtained.

### Electrochemical Characterization

Ammonia oxidation reaction (AOR) activity of Pt‐DG‐0.1, Pt‐DG‐0.5, Pt‐DG‐1, Pt‐DG‐2, Pt‐DG‐4, and Pt/C electrocatalysts were measured in 0.1 m NH_4_OH + 1 m KOH as electrolyte, respectively. CVs were recorded between 0.0 and 1.2 V versus RHE at a scanning rate of 20 mV s^−1^ at 25 to 80 °C. The ink was obtained after sonication of ethanol (950 µL) with the catalyst (2 mg) and 5 wt.% Nafion solution (50 µL), and then the dispersion was loaded on the carbon paper. The ink was dried in air and served as the work electrode. The counter and reference electrodes were a carbon rod and Hg/HgO electrode, respectively. The E (RHE) = E (Hg/HgO) + 0.059 × pH + 0.098 V in 1.0 m KOH (pH = 13.8). Ar‐saturated 1.0 m KOH was used as the electrolyte.

The Oxygen Reduction Reaction (ORR) activity tests were carried out in O_2_‐saturated 0.1 m KOH at a scan rate of 5 mV s^−1^. A polished round disk electrode (RDE) (diameter: 5.0 mm) was used as the working electrode. Before the drop‐cast of the catalysts, 2.5 mg of catalysts were dispersed into a homogeneous 950 µL blended ethanol solution with 50 µL of Nafion solution (5 wt.%) under ultrasonication. The loaded dose of the as‐prepared ink was fixed to obtain a constant loading of 0.125 mg cm^−2^.

The DAFCs experiments took place in a single cell with an area of 5 cm^−2^. The temperature was set to 40 and 95 °C for the fuel cell. The electrocatalyst was painted over carbon paper in the form of a homogeneous dispersion prepared using Nafion solution. The membranes were first exposed to 6 mol L^−1^ KOH for 24 h. The default testing condition was anode feed: 4.0 mL min^−1^ (controlled by peristaltic pump), cathode feed: O_2_, ambient pressure. All the polarization curves were obtained using a potentiostat/galvanostat (PGSTAT 302NAutolab) from Metrohm AG.

### Materials Characterization

Powder X‐ray diffraction (XRD) data of the samples were collected on a Bruker D8QUEST diffractometer equipped with mirror‐monochromatized Cu Kα radiation (λ = 0.15406 nm). Inductively coupled plasma‐optical emission spectroscopy (ICP‐OES) was using Aglient ICPOES‐730. In situ Raman spectra were measured by the Horiba LabRam 352 Odyssey Nano Raman Spectrometer system. Measurements were performed using a custom‐made in situ cell, and Au@SiO_2_ was used for surface‐enhanced Raman scattering for SHINERS experiments. X‐ray Photoelectron Spectroscopy (XPS) measurements were run on a Thermo escalab 250Xi instrument. Transmission electron microscopy (TEM) images and High‐angle annular dark field (HAADF) scanning transmission electron microscopy (STEM) images were performed by Hitachi‐HF5000. The X‐ray absorption spectroscopy (XAS) measurements for the Pt catalysts were measured at the Taiwan Light Source beamline of the National Synchrotron Radiation Research Center.

## Conflict of Interest

The authors declare no conflict of interest.

## Author Contributions

T.W., X.W., and Q.Y. contributed equally to this work. Y.L. and Z.W. supervised the project. Y.L. and T.W. conceived the idea and designed the experiments. T.W. carried out all the experimental work. X.W. and Z.W performed and supervised the computational work, respectively. Q.Y. and S.W. carried out the catalyst synthesis. T.W. and F.H. performed and supervised the catalyst characterization respectively. S.C., L.C., R.Y., and T.W. performed and analysed the XAS experiments respectively. M.Z. carried out the XPS measurements. B.W. and T.W. performed the Raman spectroscopy. T.W., X.W., Z.W., and Y.L. co‐wrote the manuscript. All authors discussed the results and assisted during the manuscript preparation.

## Supporting information



Supporting Information

## Data Availability

The data that support the findings of this study are available from the corresponding author upon reasonable request.
