# Supplementary material for: Modulating Single‐Atom Pt Coordination for Enhanced Low‐Temperature Ammonia Fuel Cell Electrocatalysis
Source: Adv Mater. 2025 Jul 26;37(41):e08371. doi: 10.1002/adma.202508371 (PMC12531743; doi:10.1002/adma.202508371)
Supplement: Supplementary file 1 — Supporting Information [file ADMA-37-e08371-s001.pdf]

ADVANCED MATERIALS

Supporting Information

for *Adv. Mater.*, DOI 10.1002/adma.202508371

Modulating Single-Atom Pt Coordination for Enhanced Low-Temperature Ammonia Fuel Cell Electrocatalysis

Tong Wu, Xingyu Wang, Qin Yang, Bingqing Wang, Ruoou Yang, Shin-An Chen, Lo-Yueh Chang, Sibo Wang, Fuqiang Huang, Ziyun Wang* and Yanwei Lum**

Supporting Information

Modulating Single-Atom Pt Coordination for Enhanced Low-Temperature Ammonia Fuel Cell Electrocatalysis

Tong Wu[1,2,7], Xingyu Wang[3,7], Qin Yang[1,7], Bingqing Wang[1,2], Ruoou Yang[1], Shin-An Chen[4], Lo-Yueh Chang[4], Sibo Wang[1,2], Fuqiang Huang[5]*, Ziyun Wang[3]* and Yanwei Lum[1,2,6]*

[1]Department of Chemical and Biomolecular Engineering, National University of Singapore, Singapore, 117585, Republic of Singapore

[2]Centre for Hydrogen Innovations, National University of Singapore, Singapore, 117580, Republic of Singapore

[3]School of Chemical Sciences, The University of Auckland, Auckland, New Zealand

[4]National Synchrotron Radiation Research Center, Hsinchu, 30076, Taiwan

[5]State Key Lab of Metal Matrix Composites, School of Materials Science and Engineering, Shanghai Jiao Tong University, Shanghai, 200240, China

[6]Institute of Materials Research and Engineering, Agency for Science, Technology and Research (A*STAR), 2 Fusionopolis Way, Innovis #08-03, Singapore, 138634, Republic of Singapore

[7]These authors contributed equally to this work.

*Corresponding author: huangfq@sjtu.edu.cn

*Corresponding author: ziyun.wang@auckland.ac.nz

*Corresponding author: lumyw@nus.edu.sg

Computational method

Computational investigations were performed employing the Vienna Ab-initio Simulation Package (VASP) using density functional theory (DFT). [1] We implemented the Perdew-Burke-Ernzerhof (PBE) functional within the generalized gradient approximation (GGA) to capture electron exchange and correlation energetics.[2] The core-valence electron interactions were modelled through the frozen-core projector augmented-wave (PAW) methodology, establishing an energy cutoff of 450 eV.[3]

Geometric optimization followed stringent convergence protocols: energy criteria of 1.0×10^{-5} eV per atom and force constraints of 0.05 eV $Å^{-1}$. Structural refinements utilized a Γ-centered Monkhorst-Pack k-point mesh configured as 3×3×1.[4] Bader charge analysis enabled detailed exploration of charge transformations for $*NH_2$ and $*NH_2NH_2$ species within Pt-5N2Cl-2 structural configurations.[5]

The intermediate free energy formulation incorporates multifaceted energetic contributions: ΔE represents the stepwise reaction energies derived from DFT calculations; ΔE_{ZPE} quantifies zero-point energy variations across reaction pathways; TΔS accounts for entropic contributions at 298.15 K, capturing the entropy differential between adsorbed and gaseous states. Consistent with the computational hydrogen electrode (CHE) methodology, a proton/electron pair's energy is defined as half the hydrogen molecule's energy.[6]

The adsorption energy (ΔE_{ads}) of the key intermediates, including $*NH_3$, $*NH_2$, $*NH_2NH_2$, *NHNH, $*NNH_2$, *NNH, was calculated relative to NH_3 and H_2 under

conditions of T = 298.15 K according to following equations:

$$\Delta E_{*NH_3} = E_{*NH_3} - E_{NH_3} - E^* \quad (1)$$

$$\Delta E_{*NH_2} = E_{*NH_2} - E_{NH_3} - E^* + 1/2E_{H_2} \quad (2)$$

$$\Delta E_{*NH_2NH_2} = E_{*NH_2NH_2} - 2E_{NH_3} - E^* + E_{H_2} \quad (3)$$

$$\Delta E_{*NHNH_2} = E_{*NHNH_2} - 2E_{NH_3} - E^* + 3/2E_{H_2} \quad (4)$$

$$\Delta E_{*NNH_2} = E_{*NNH_2} - 2E_{NH_3} - E^* + 2E_{H_2} \quad (5)$$

$$\Delta E_{*NNH} = E_{*NNH} - 2E_{NH_3} - E^* + 5/2E_{H_2} \quad (6)$$

The Gibbs free energy changes (ΔG) for all intermediates were then calculated from the electronic energies by incorporating entropy and zero-point energy corrections[6]:

$$\Delta G = \Delta E - \Delta ZPE - T\Delta S \quad (7)$$

For the two key steps mentioned above - $*NH_2$ formation and $*NH_2NH_2$ coupling - the calculation formulas are as follows:

$$\Delta G_{(*NH_2 + NH_3 \to *NH_2NH_2 + 1/2H_2)} = G_{*NH_2NH_2} - G_{NH_3} - G_{*NH_2} + 1/2G_{H_2} \quad (8)$$

$$\Delta G_{(*NH_3 \to *NH_2 + 1/2H_2)} = G_{*NH_2} - G_{NH_3} + 1/2G_{H_2} \quad (9)$$

The equilibrium potential of U= 0.7 V has been added to each equation involving hydrogen, reflecting the electrochemical conditions of the reaction.

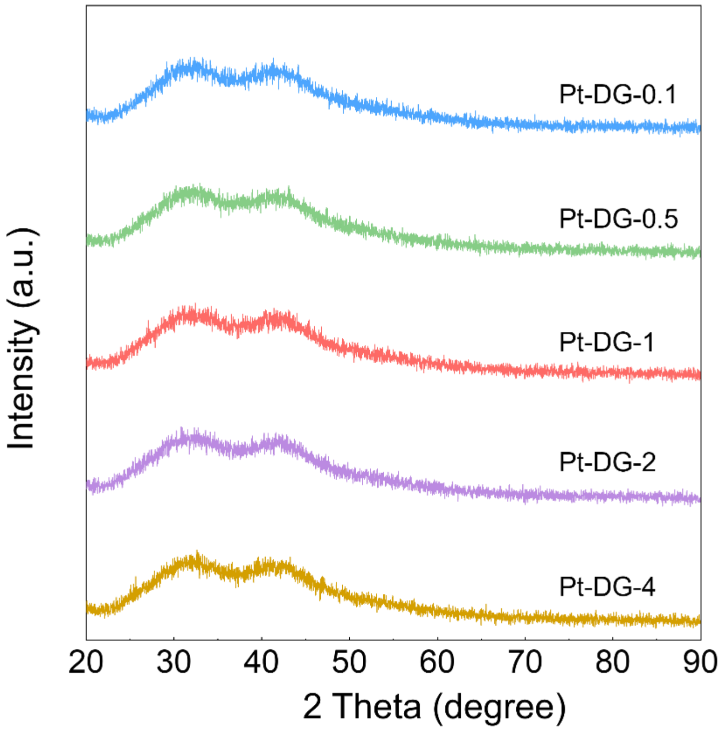

Figure S1. XRD patterns of Pt-DG-0.1, Pt-DG-0.5, Pt-DG-1, Pt-DG-2, and Pt-DG-4.

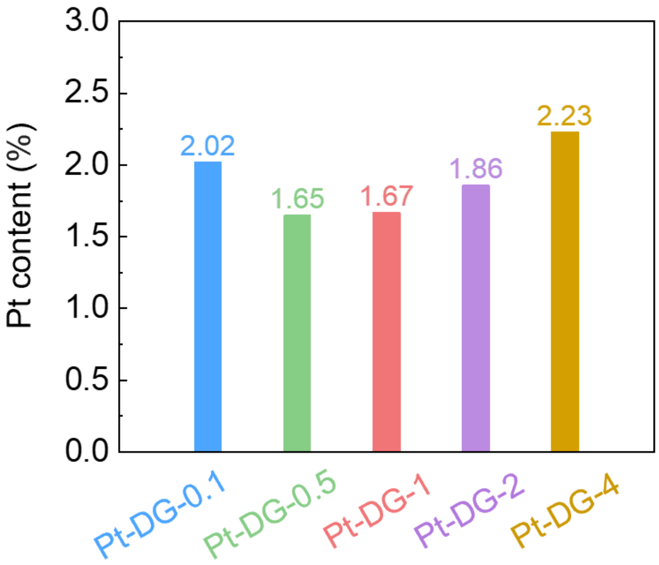

Figure S2. The Pt content (by weight) measured using inductively coupled plasma atomic emission spectroscopy (ICP-OES) of Pt-DG-0.1, Pt-DG-0.5, Pt-DG-1, Pt-DG-2, and Pt-DG-4.

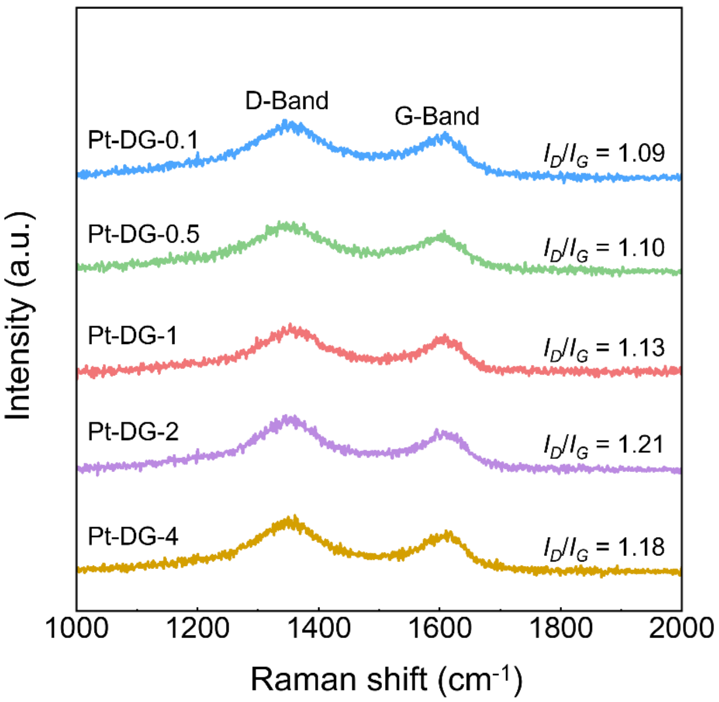

Figure S3. Raman spectra of Pt-DG-0.1, Pt-DG-0.5, Pt-DG-1, Pt-DG-2, and Pt-DG-4.

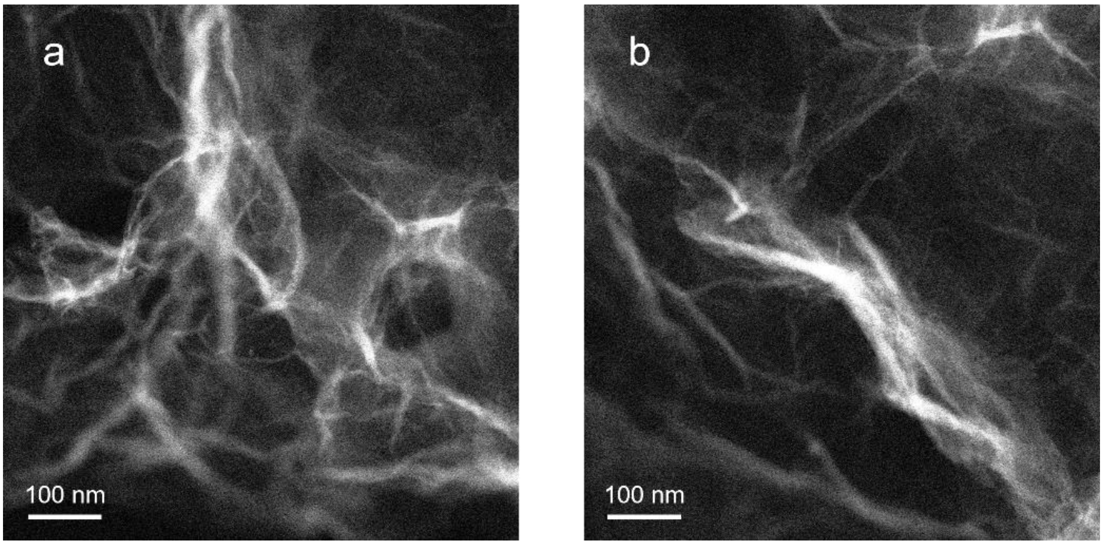

Figure S4. Wider field-of-view TEM images of the as obtained Pt-DG-1 sample after the photoreduction process.

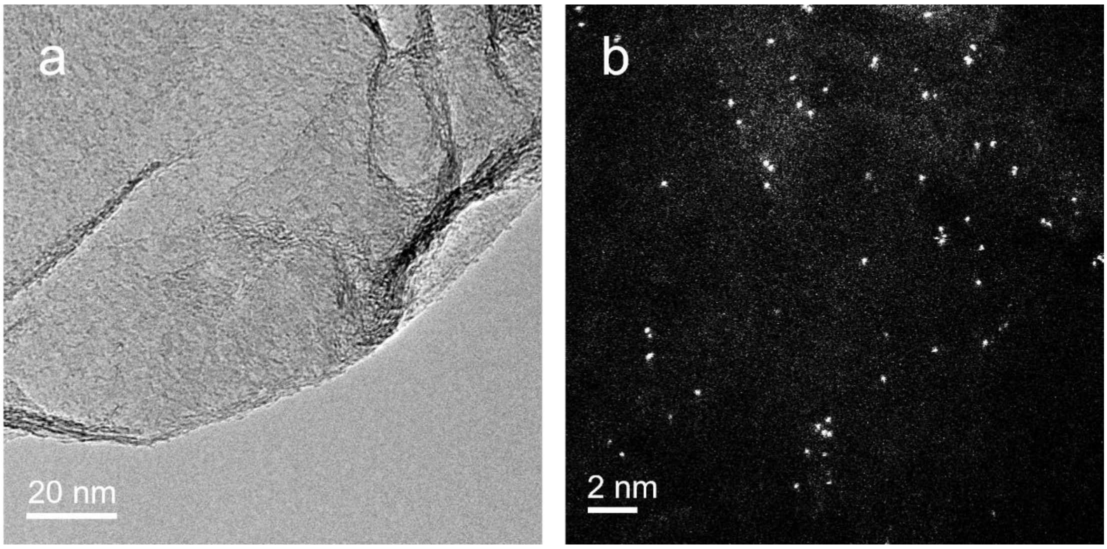

Figure S5. a) TEM and b) aberration-corrected high-angle annular dark-field STEM images of Pt-DG-0.1.

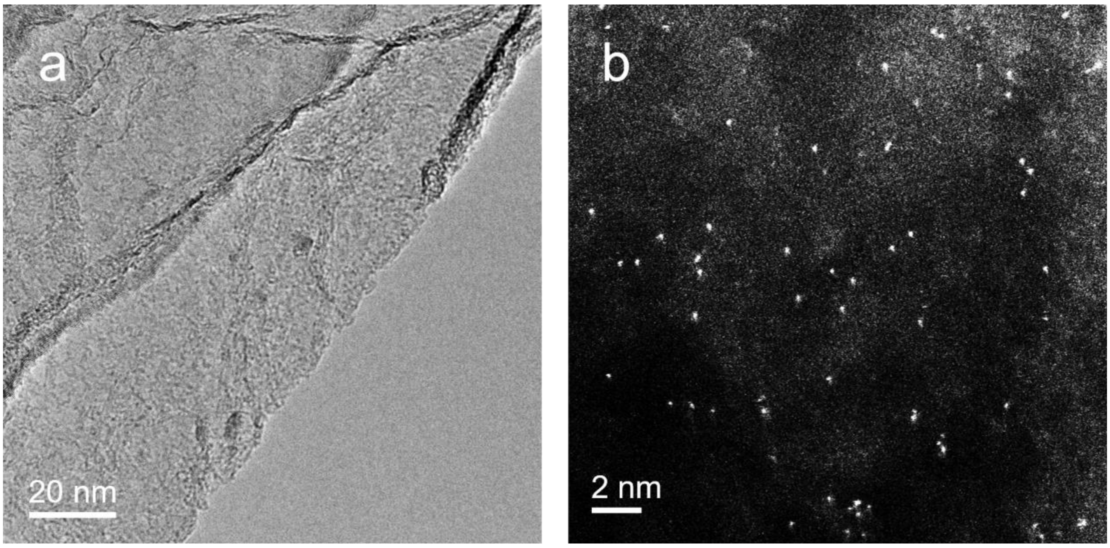

Figure S6. a) TEM and b) aberration-corrected high-angle annular dark-field STEM images of Pt-DG-0.5.

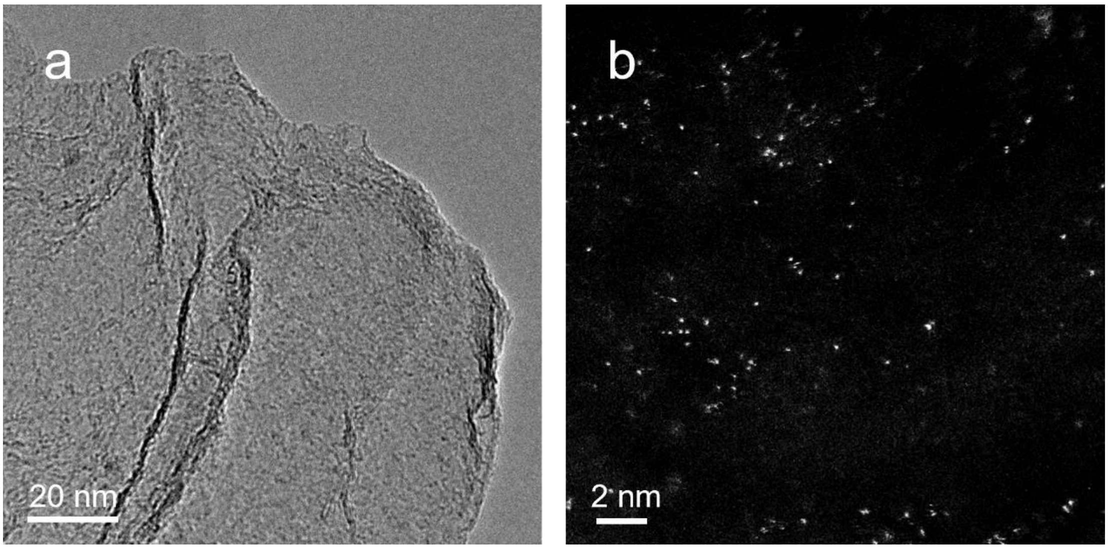

Figure S7. a) TEM and b) aberration-corrected high-angle annular dark-field STEM images of Pt-DG-2.

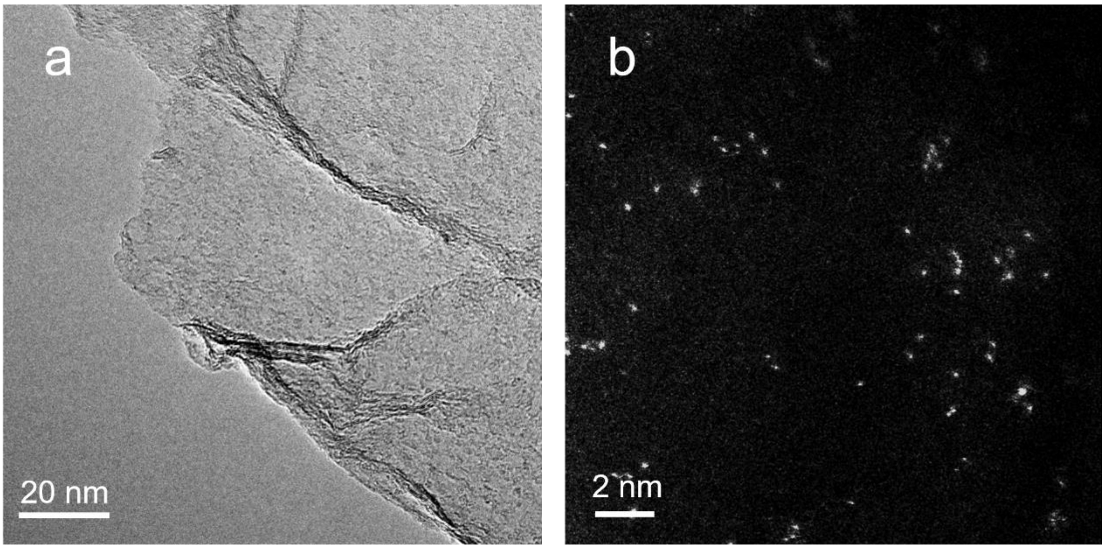

Figure S8. a) TEM and b) aberration-corrected high-angle annular dark-field STEM images of Pt-DG-4.

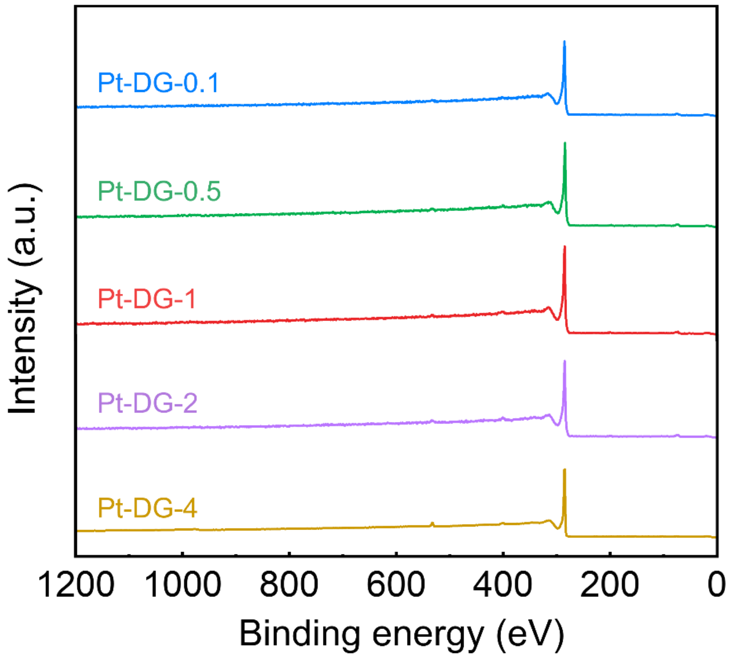

Figure S9. The XPS survey spectra of Pt-DG-0.1, Pt-DG-0.5, Pt-DG-1, Pt-DG-2, and Pt-DG-4.

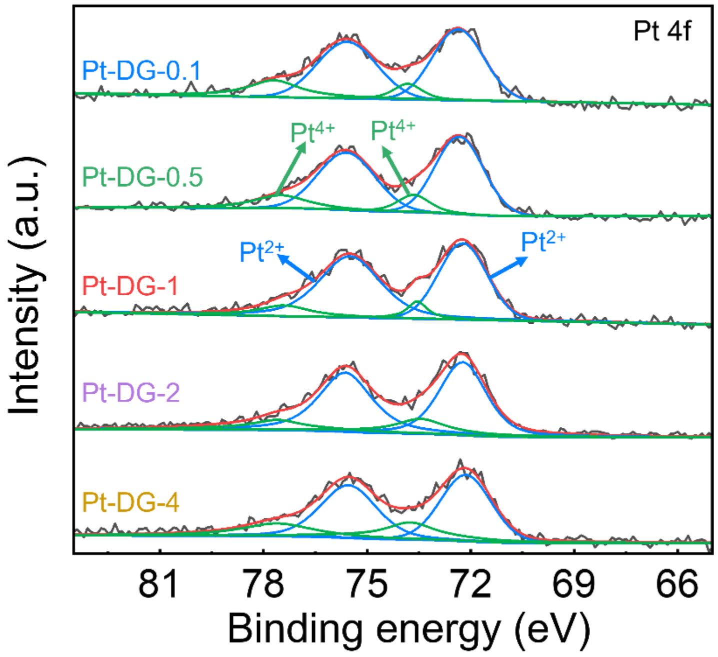

Figure S10. High-resolution Pt 4f XPS spectra of Pt-DG-0.1, Pt-DG-0.5, Pt-DG-1, Pt-DG-2, and Pt-DG-4.

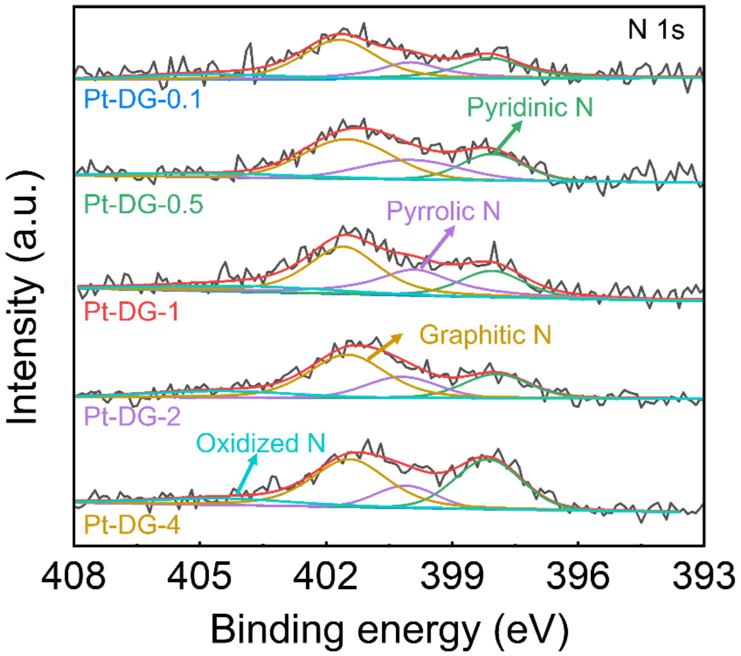

Figure S11. High-resolution N 1s XPS spectra of Pt-DG-0.1, Pt-DG-0.5, Pt-DG-1, Pt-DG-2, and Pt-DG-4.

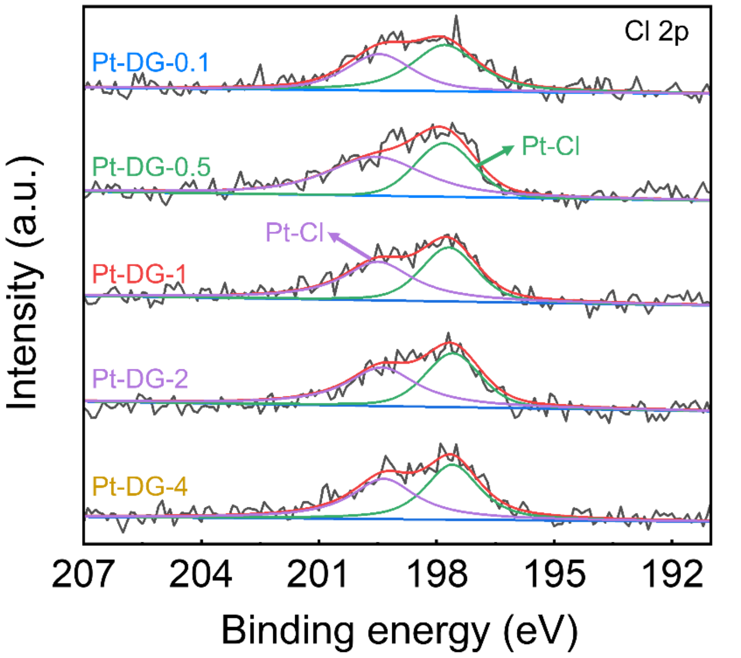

Figure S12. High-resolution Cl 2p XPS spectra of Pt-DG-0.1, Pt-DG-0.5, Pt-DG-1, Pt-DG-2, and Pt-DG-4.

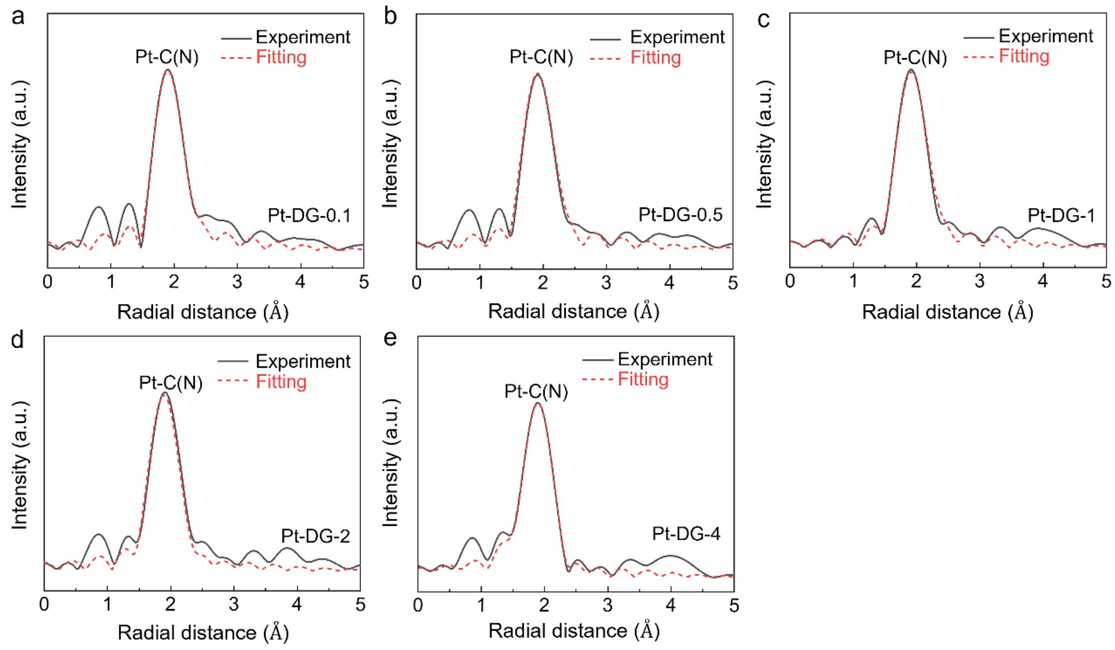

Figure S13. EXAFS fitting in R-space for a) Pt-DG-0.1, b) Pt-DG-0.5, c) Pt-DG-1, d) Pt-DG-2, and e) Pt-DG-4.

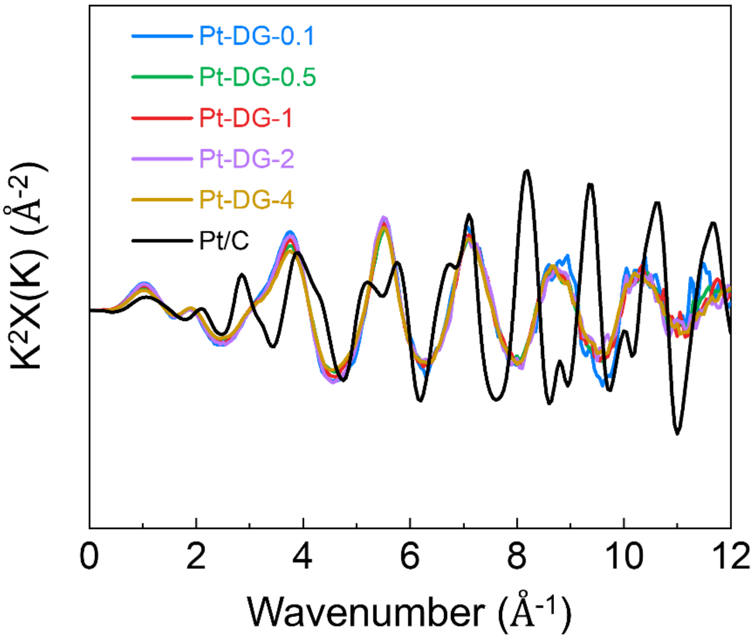

Figure S14. The Pt L-edge EXAFS oscillations of Pt-DG-0.1, Pt-DG-0.5, Pt-DG-1, Pt-DG-2, Pt-DG-4, and Pt/C.

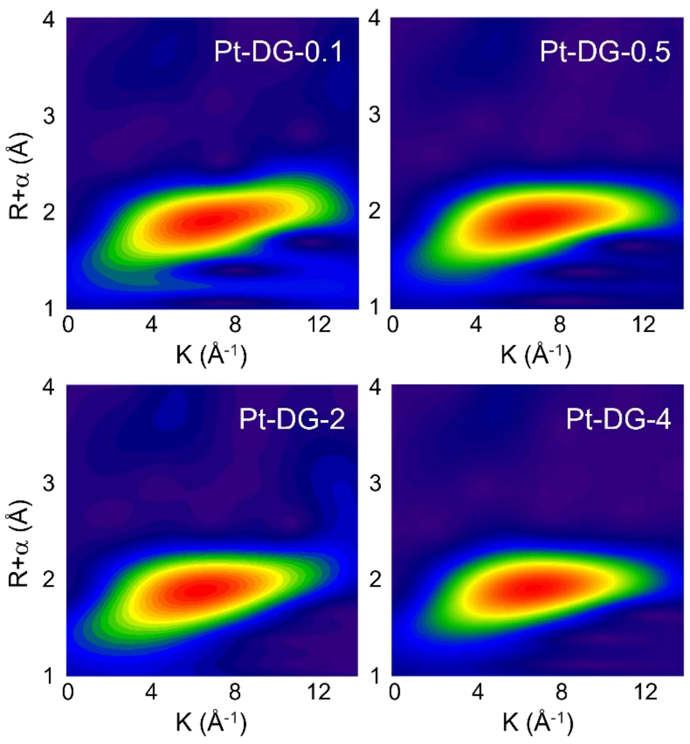

Figure S15. Wavelet transforms for the k2-weighted Pt L-edge EXAFS of Pt-DG-0.1, Pt-DG-0.5, Pt-DG-2, and Pt-DG-4.

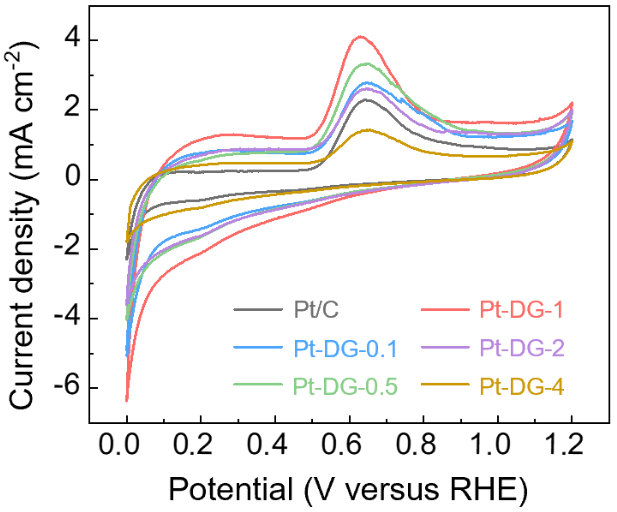

Figure S16. AOR CV curves of Pt-DG-x and Pt/C obtained using 0.1 M NH_4OH + 1.0 M KOH electrolyte without mass normalization.

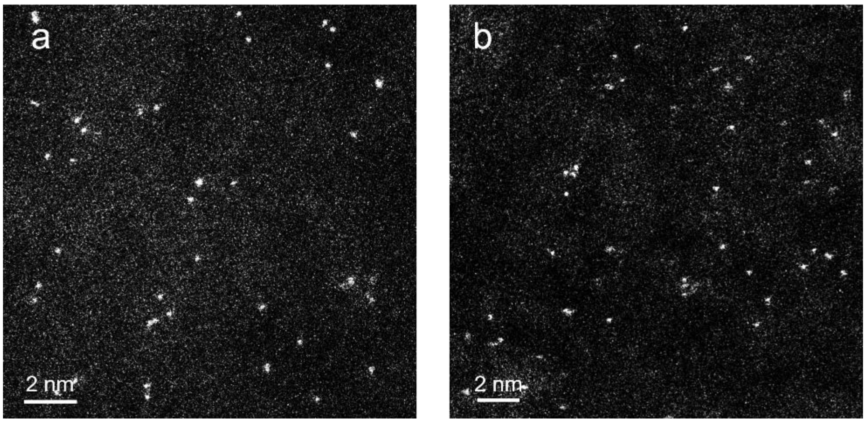

Figure S17. The aberration-corrected high-angle annular dark-field STEM images of Pt-DG-1 after AOR.

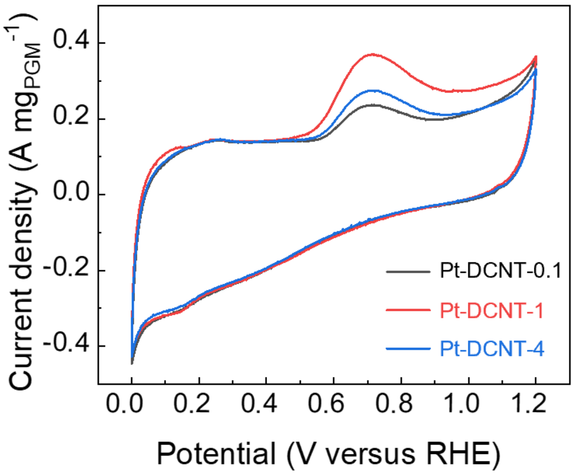

Figure S18. AOR CV curves of Pt-DCNT-x obtained using 0.1 M NH_4OH + 1.0 M KOH.

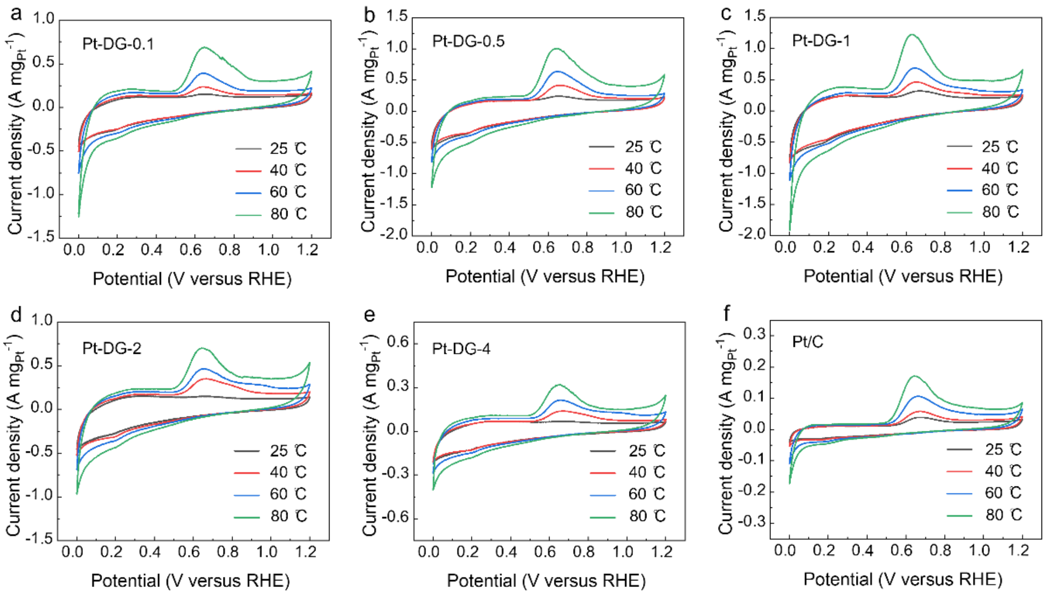

Figure S19. The CV curves of a) Pt-DG-0.1, b) Pt-DG-0.5, c) Pt-DG-1, d) Pt-DG-2, e) Pt-DG-4, and f) Pt/C in Ar-saturated 1.0 M KOH + 0.1 M NH_4OH at different temperatures.

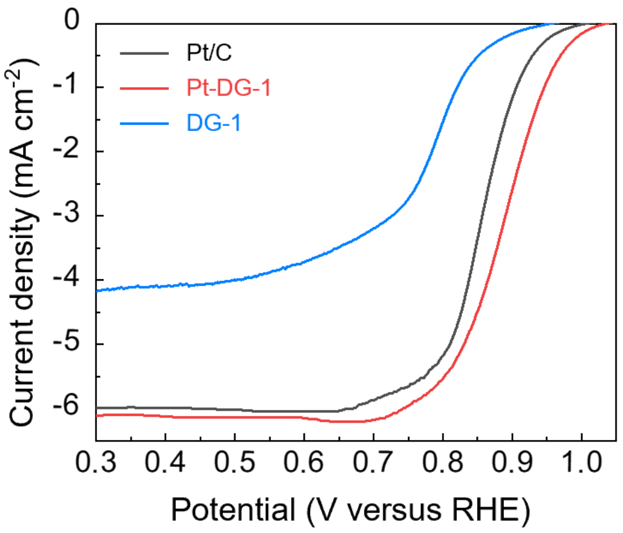

Figure S20. ORR polarization plots of Pt-DG-1, DG-1, and Pt/C in 0.1 M KOH.

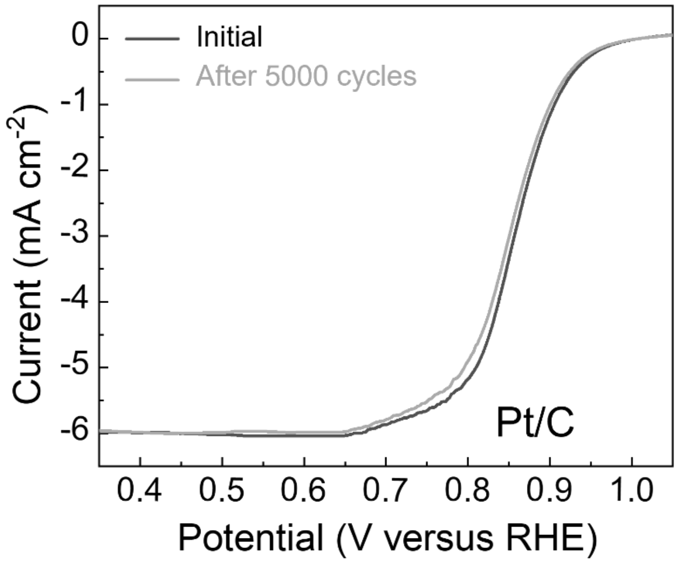

Figure S21. ORR polarization curves before and after 5000 cycles of Pt/C in 0.1 M KOH.

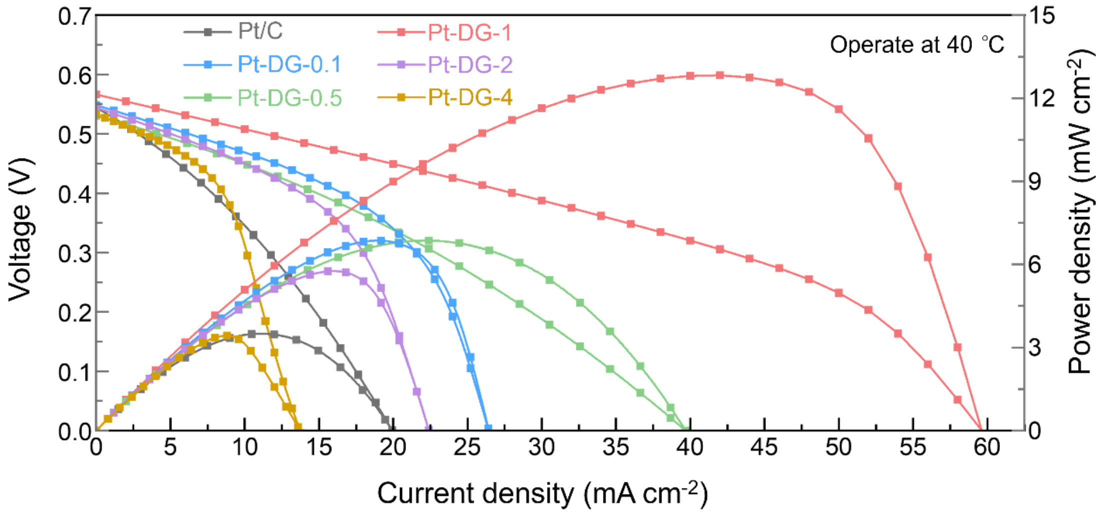

Figure S22. Polarization and power density curves of a 5 cm^2 DAFC at 40 °C for Pt-DG-0.1, Pt-DG-0.5, Pt-DG-1, Pt-DG-2, Pt-DG-4, and Pt/C in 5 M NH_4OH and 1 M KOH.

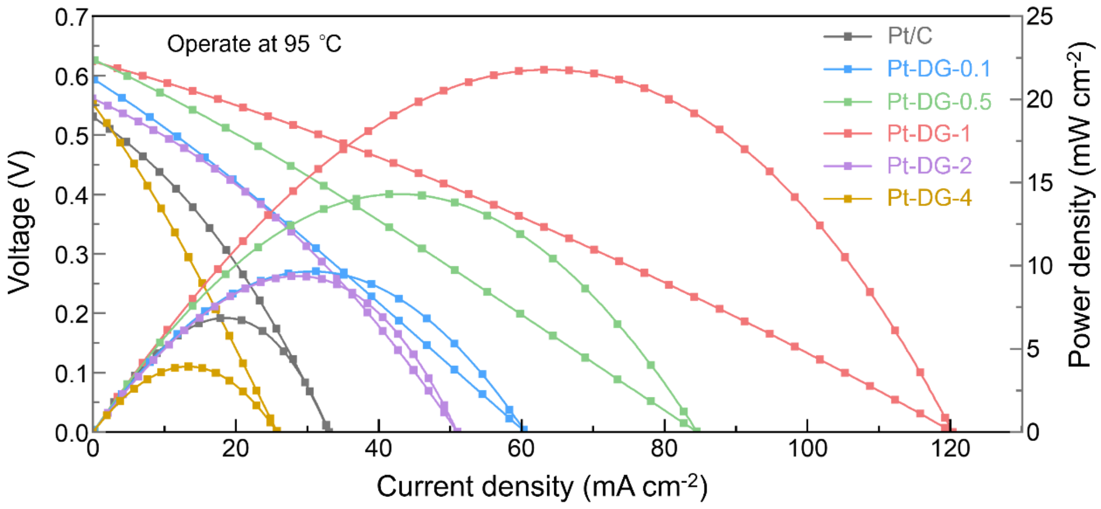

Figure S23. Polarization and power density curves of a 5 cm^2 DAFC at 95 °C for Pt-DG-0.1, Pt-DG-0.5, Pt-DG-1, Pt-DG-2, Pt-DG-4, and Pt/C in 5 M NH_4OH and 1 M KOH.

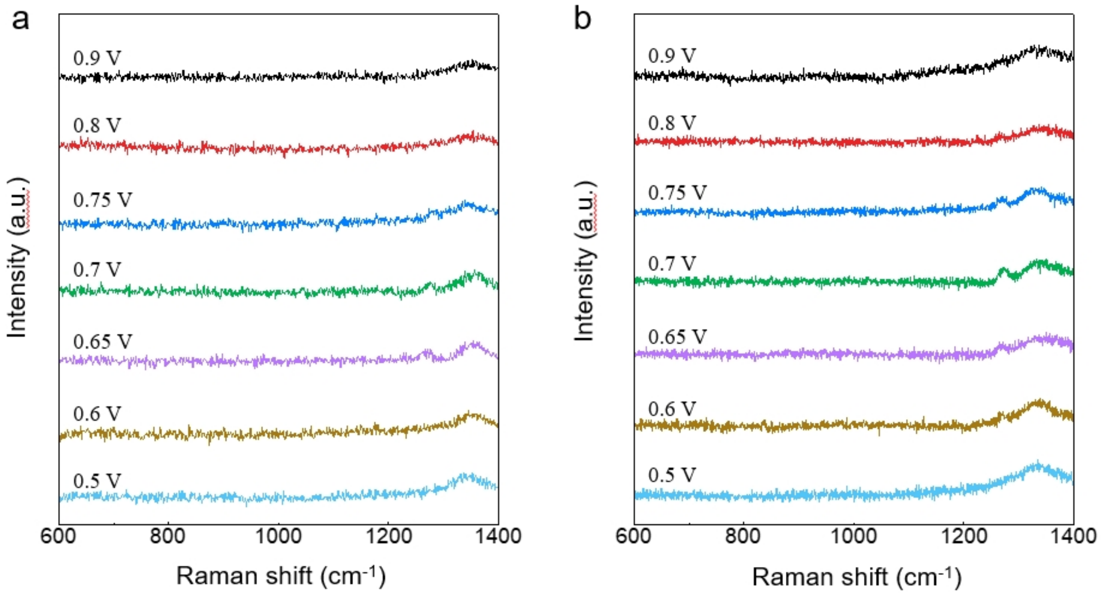

Figure S24. The in-situ Raman spectra of a) Pt-DG-1 and b) Pt/C during the AOR process from 0.5 to 0.9 V vs RHE in N_2-saturated 1.0 M KOH + 0.1 M NH_4OH.

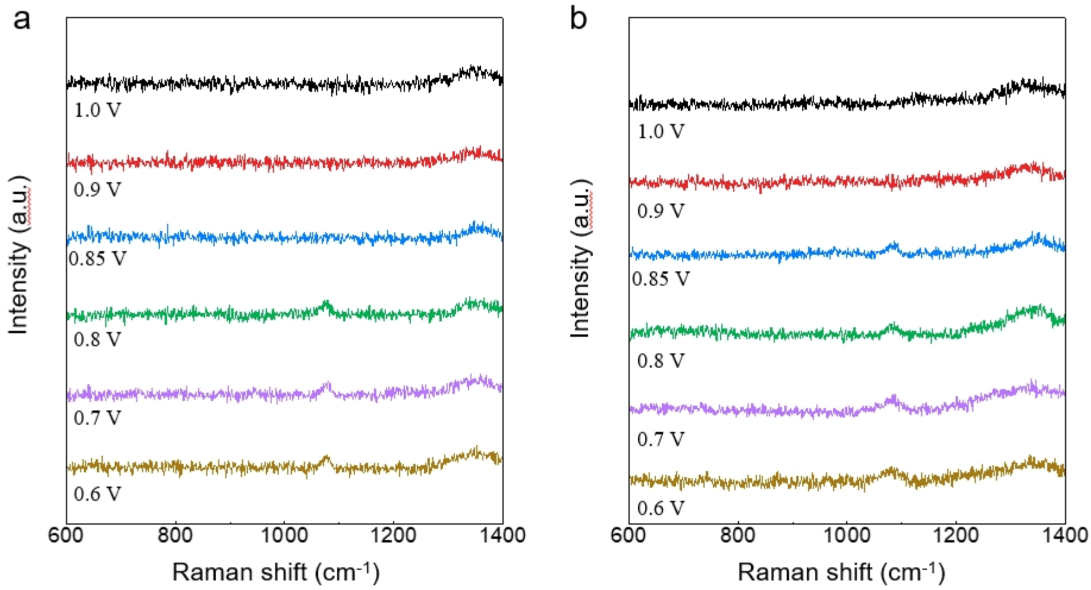

Figure S25. The in-situ Raman spectra of a) Pt-DG-1 and b) Pt/C during the ORR process from 1.0 to 0.6 V vs RHE in O_2-saturated 0.1 M KOH.

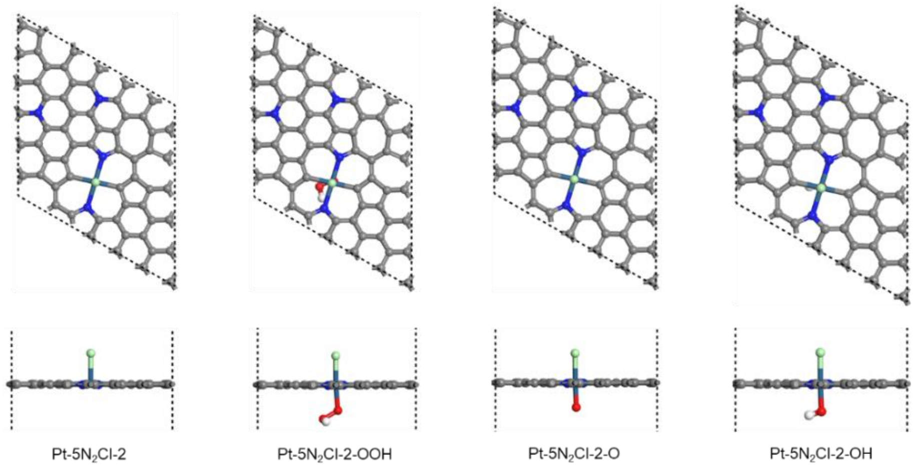

Figure S26. DFT calculations of OH coverage effects on ORR activity. Top and side views of Pt-5N_2Cl-2 with different ORR intermediates: pure surface, *OOH, *O, and *OH adsorbed configurations.

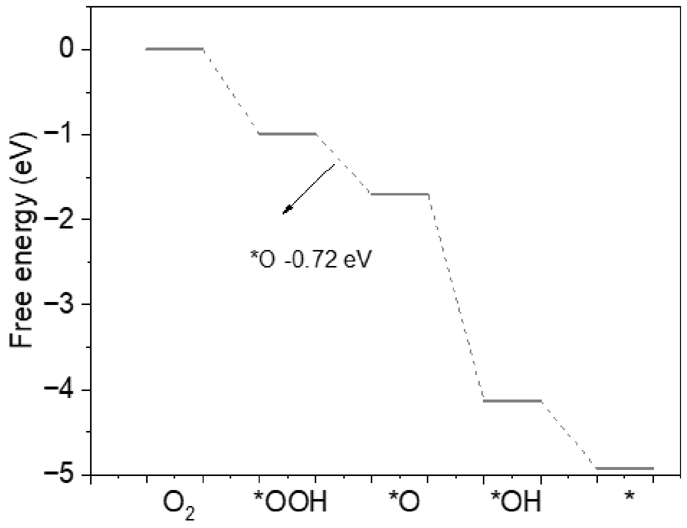

Figure S27. Free energy diagram for ORR on Pt-5N_2Cl-2.

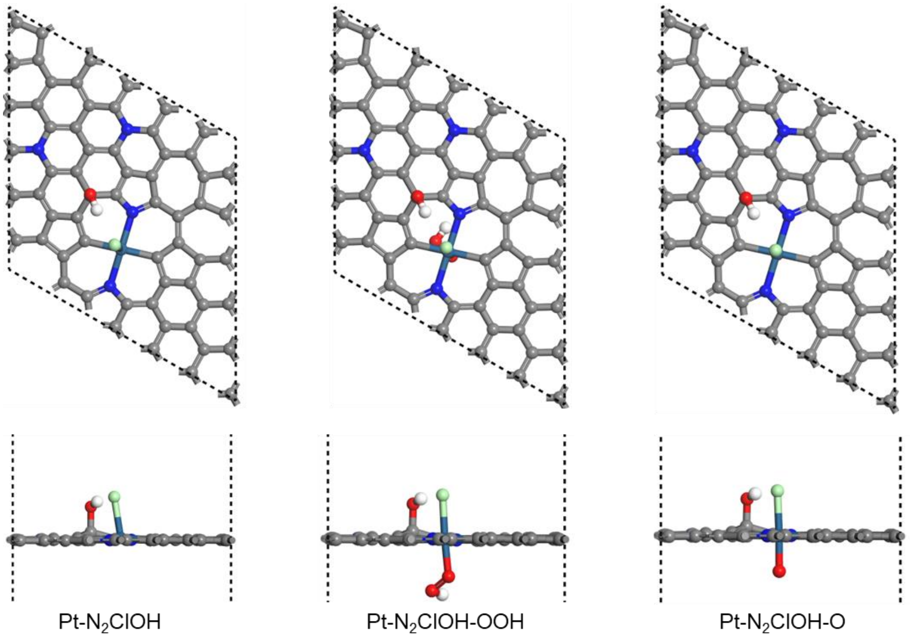

Figure S28. Top and side views of Pt-N_2ClOH (with pre-adsorbed *OH) showing *OOH and *O intermediates.

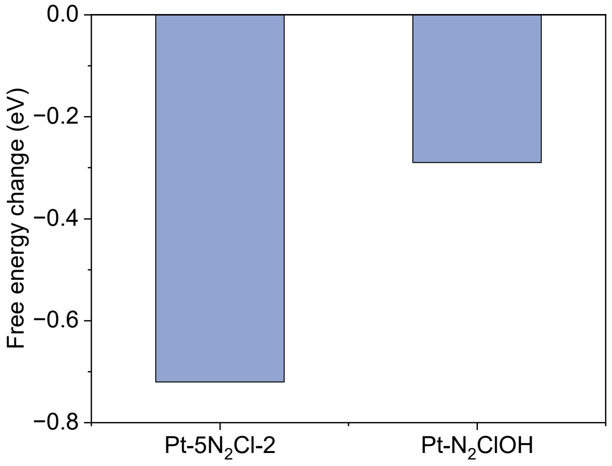

Figure S29. Comparison of PDS free energy changes between Pt-5N_2Cl-2 and *OH-covered Pt-N_2ClOH site.

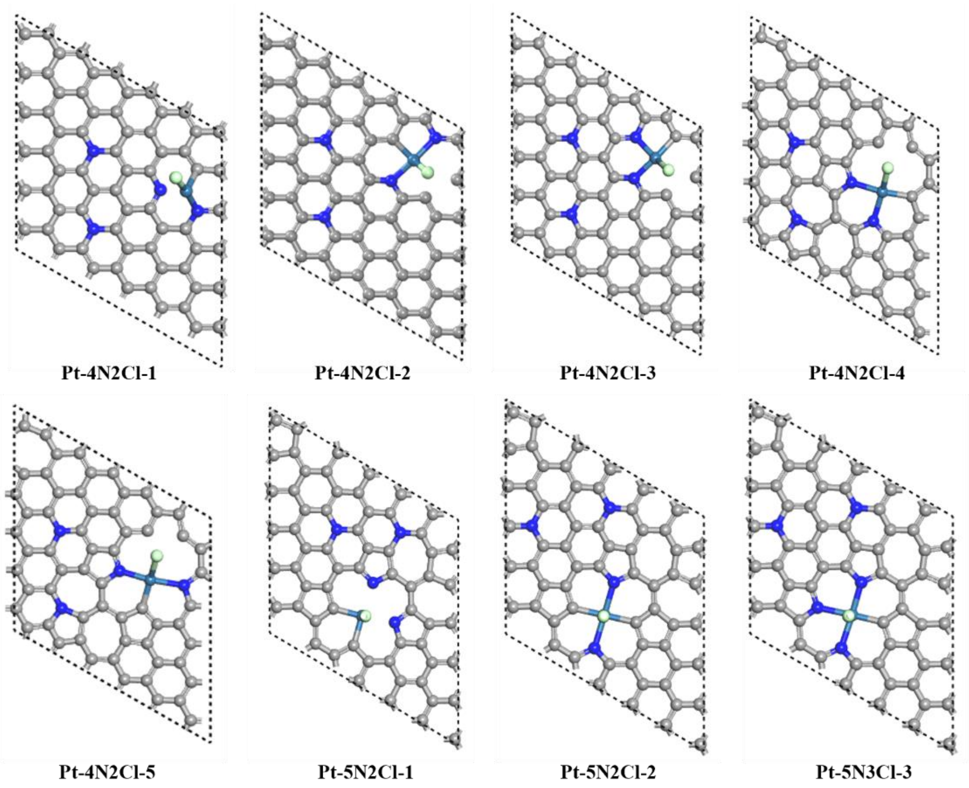

Figure S30. Structural configurations of various single-atom catalysts: Pt-4N2Cl-1, Pt-4N2Cl-2, Pt-4N2Cl-3, Pt-4N2Cl-4, Pt-4N2Cl-5, Pt-5N2Cl-1, Pt-5N2Cl-2, and Pt-5N2Cl-3.

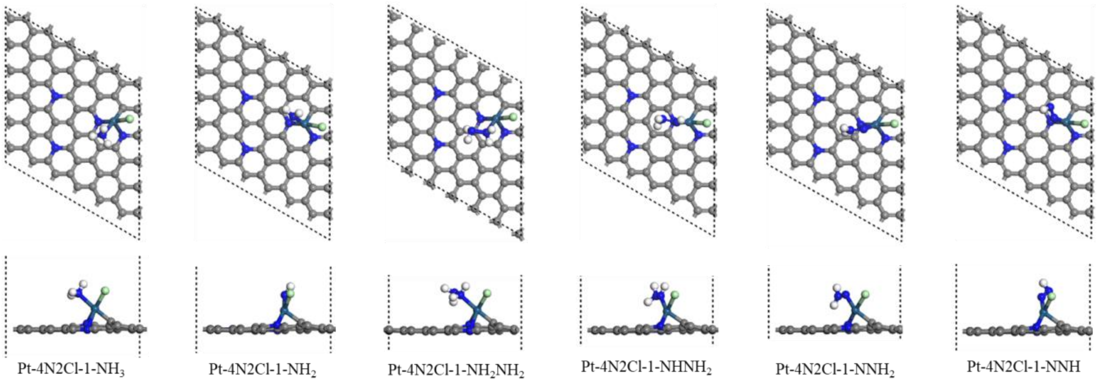

Figure S31. The top and side views of Pt-4N2Cl-1 with adsorbed *NH_3, *NH_2, *NH_2NH_2, *$NHNH_2$, *NNH_2, *NNH. The white, blue, grey and green spheres represent H, N, C and Cl atoms respectively.

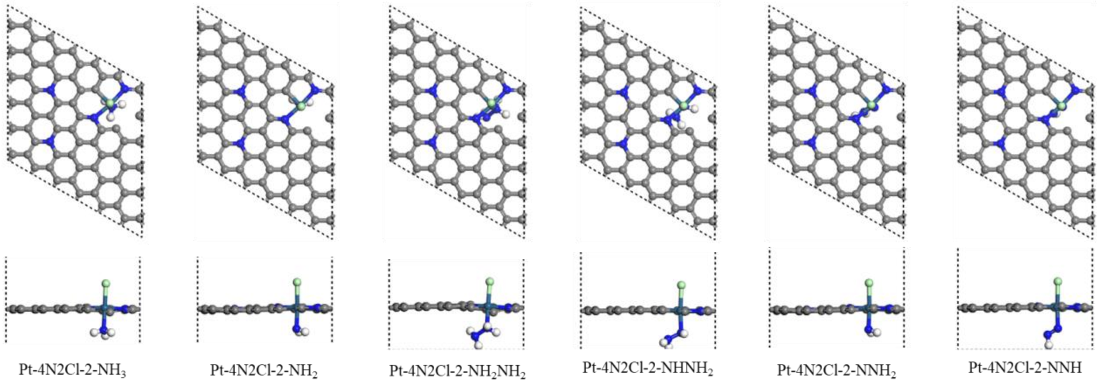

Figure S32. The top and side views of Pt-4N2Cl-2 with adsorbed *NH_3, *NH_2, *NH_2NH_2, *$NHNH_2$, *NNH_2, *NNH. The white, blue, grey and green spheres represent H, N, C and Cl atoms respectively.

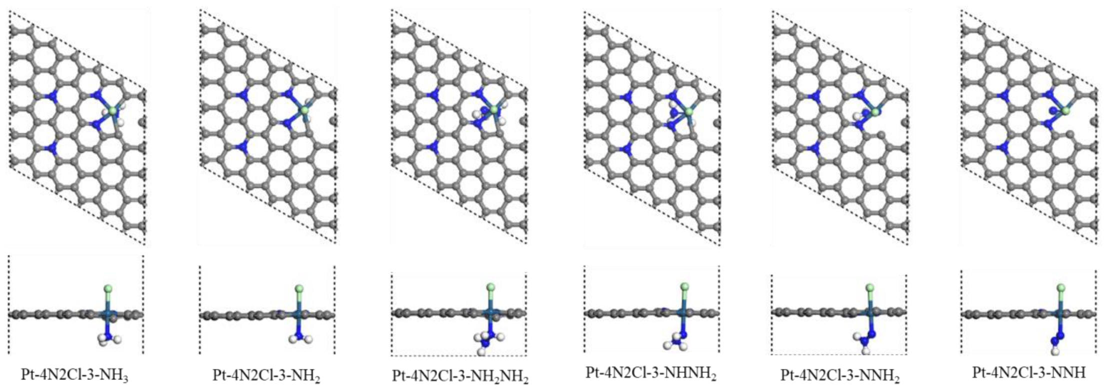

Figure S33. The top and side views of Pt-4N2Cl-3 with adsorbed *NH_3, *NH_2, *NH_2NH_2, *$NHNH_2$, *NNH_2, *NNH. The white, blue, grey and green spheres represent H, N, C and Cl atoms respectively.

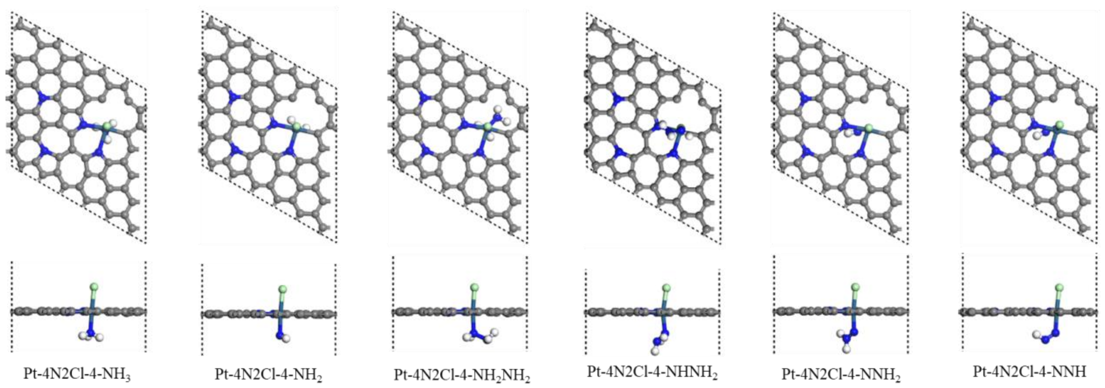

Figure S34. The top and side views of Pt-4N2Cl-4 with adsorbed *NH_3, *NH_2, *NH_2NH_2, *$NHNH_2$, *NNH_2, *NNH. The white, blue, grey and green spheres represent H, N, C and Cl atoms respectively.

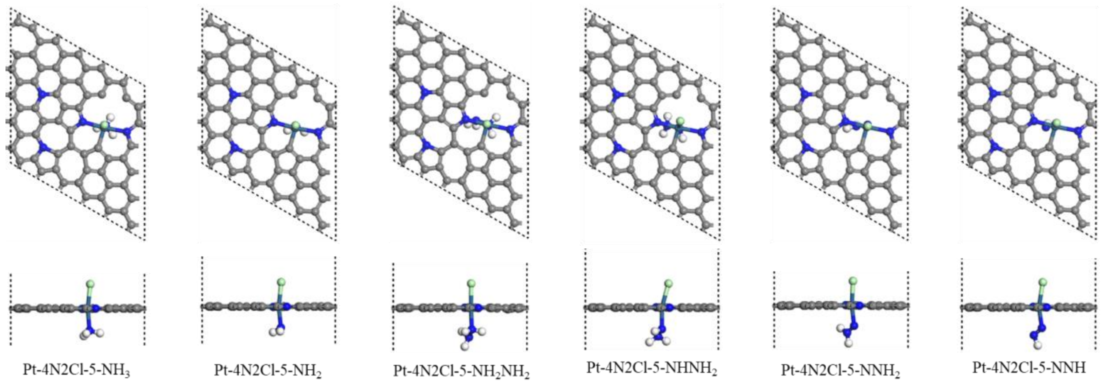

Figure S35. The top and side views of Pt-4N2Cl-5 with adsorbed $*NH_3$, $*NH_2$, $*NH_2NH_2$, $*NHNH_2$, $*NNH_2$, *NNH. The white, blue, grey and green spheres represent H, N, C and Cl atoms respectively.

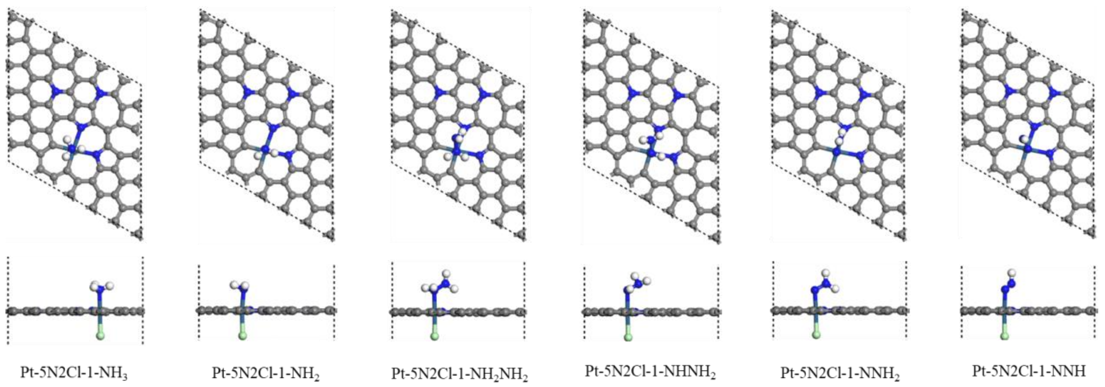

Figure S36. The top and side views of Pt-5N2Cl-1 with adsorbed $*NH_3$, $*NH_2$, $*NH_2NH_2$, $*NHNH_2$, $*NNH_2$, *NNH. The white, blue, grey and green spheres represent H, N, C and Cl atoms respectively.

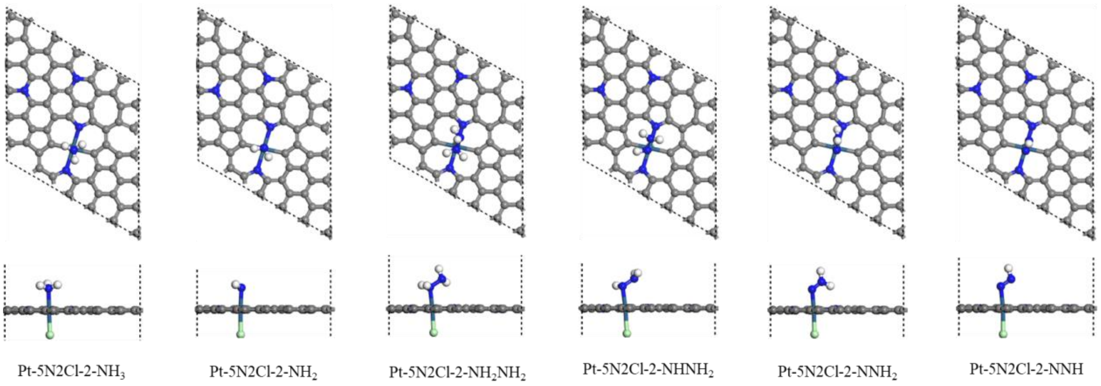

Figure S37. The top and side views of Pt-5N2Cl-2 with adsorbed *NH_3, *NH_2, *NH_2NH_2, *$NHNH_2$, *NNH_2, *NNH. The white, blue, grey and green spheres represent H, N, C and Cl atoms respectively.

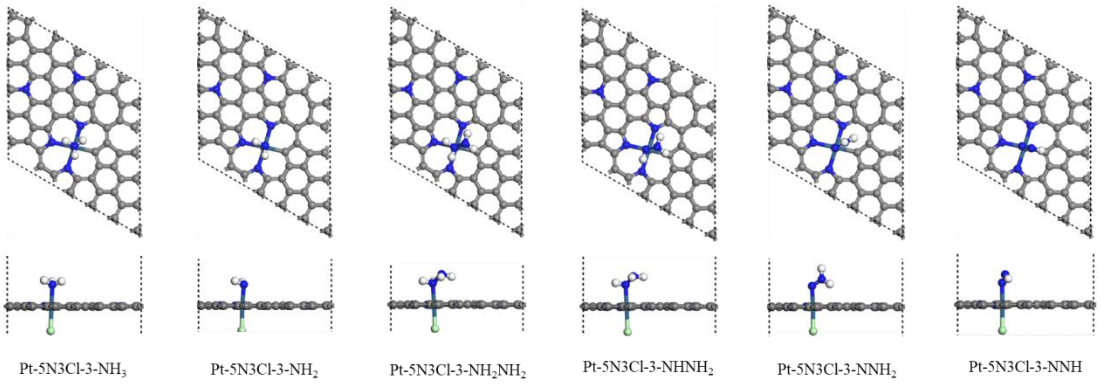

Figure S38. The top and side views of Pt-5N2Cl-3 with adsorbed *NH_3, *NH_2, *NH_2NH_2, *$NHNH_2$, *NNH_2, *NNH. The white, blue, grey and green spheres represent H, N, C and Cl atoms respectively.

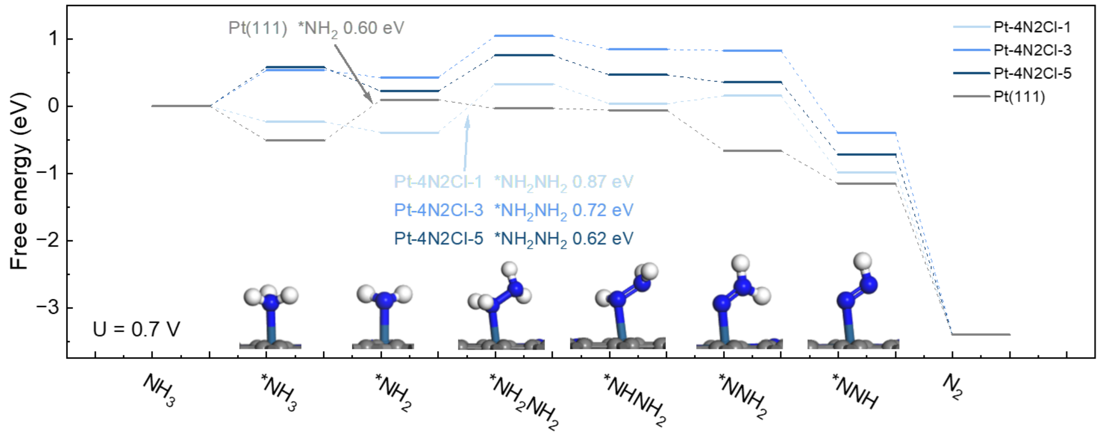

Figure S39. The free energy diagram showing the pathway of NH_3 electroreduction to N_2, including intermediates $*NH_3$, $*NH_2$, $*NH_2NH_2$, $*NHNH_2$, $*NNH_2$, $*NNH$. The highlight indicates the potential determining steps of single-atom catalysts at 0.7V vs. RHE.

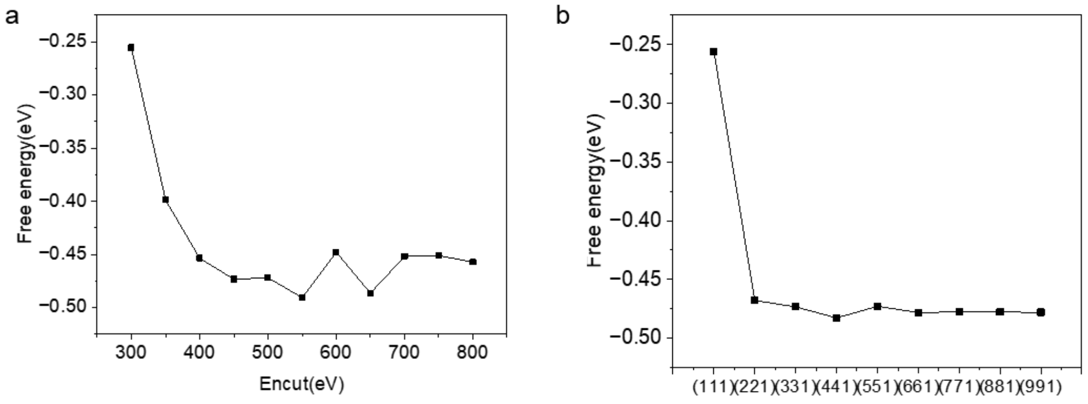

Figure S40. Convergence tests for $*NH_3$ adsorption on Pt-5N2Cl-1. a) Cutoff energy convergence test. b) K-point mesh convergence test.

For the cutoff energy, we tested values from 300 to 800 eV and found that 450 eV provides adequate convergence for our Pt-containing system, with the free energy change reaching a converged plateau beyond this point (**Figure S40**a). For K-points (**Figure S40**b), we tested meshes from 1×1×1 to 9×9×1 and confirmed that the 3×3×1 mesh yields well-converged results, with the free energy change approaching a plateau at this density compared to denser meshes. These convergence tests confirm that our computational parameters (ENCUT=450 eV, KPOINTS=3×3×1) meet the convergence criteria for accurate calculations.

Table S1. Different types of N content in Pt-DG-0.1, Pt-DG-0.5, Pt-DG-1, Pt-DG-2, and Pt-DG-4.

Catalysts	Graphitic N	Pyrrolic N	Pyridinic N	Oxidation N
Pt-DG-0.1	45.8%	20.5%	24.5%	9.2%
Pt-DG-0.5	44.8%	24.1%	21.9%	9.2%
Pt-DG-1	41.5%	29.2%	19.4%	9.9%
Pt-DG-2	48.2%	19.3%	21.3%	11.2%
Pt-DG-4	42.2%	13.1%	33.4%	11.3%

Table S2. EXAFS parameters of Pt-DG-0.1, Pt-DG-0.5, Pt-DG-1, Pt-DG-2, and Pt-DG-4.

Catalysts	Shell	CN	R (Å)	σ^2 ($Å^2$)	R-factor
Pt-DG-0.1	Pt-C(N)	3.8	2.18	0.0015	0.00557
	Pt-Cl	1.4	2.35	0.0011	
Pt-DG-0.5	Pt-C(N)	3.6	2.17	0.0017	0.00572
	Pt-Cl	1.4	2.37	0.0024	
Pt-DG-1	Pt-C(N)	3.6	2.16	0.0016	0.00504
	Pt-Cl	1.3	2.37	0.0011	
Pt-DG-2	Pt-C(N)	2.8	2.15	0.0012	0.00583
	Pt-Cl	1.4	2.35	0.0011	
Pt-DG-4	Pt-C(N)	2.7	2.15	0.0015	0.00404
	Pt-Cl	1.4	2.36	0.0012	

CN, coordination number with an error of 20%. R, distance between absorber and backscatter atoms with an error of 1%. σ^2, Debye-Waller factor with an error of 10%.

Table S3. AOR onset potential comparison for Pt-DG-x and Pt/C.

Catalysts	Pt-DG-0.1	Pt-DG-0.5	Pt-DG-1	Pt-DG-2	Pt-DG-4	Pt/C
Onset potential (vs. RHE)	0.489 V	0.473 V	0.475 V	0.484 V	0.499 V	0.522 V

Table S4. Comparison of the AOR activity of the Pt-DG-1 with some recently reported Pt-based catalysts.

Catalysts	Peak potential (V vs. RHE)	Current density (A g_{Pt}^{-1})	Reference
Pt-DG-1	0.668	322	This work
Pt/CeO_2-ZIF-8	0.682	42	Energy Environ. Sci., 2021, 14, 1449
$Pt_3Ru_{1/2}Co_{1/2}$	0.673	175	Nat. Commun., 2023, 14, 792
coral-like Pt nanowires	0.69	76	Adv. Funct. Mater. 2022, 32, 2110702
Au@Pt NPs	0.678	59	Angew. Chem. Int. Ed. 2020, 59, 18430
Pt_xRu nanocube	0.68	192	J. Mater. Chem. A, 2021, 9, 8444
Pt-NCs	0.679	135	J. Energy Chem., 2020, 47, 234
Pt/SiO_2-CNT-COOH	0.7	124	ACS Catal. 2020, 10, 3945
PtRh/C	0.71	91	Appl. Catal. B-environ., 2015, 174, 136
Pt-CNT	0.78	24	Electrochim. Acta, 2020, 341, 136027
Pt/NiO	0.67	7	Electrochim. Acta, 2016, 222, 1455

Table S5. AOR performance comparison with literature reported Pt/C catalysts.

Catalyst	mass activity	Scan rate	Reference
Pt/C	57.6 mA mg^{-1}	20 mV s^{-1}	*This work*
Pt/C	62.7 mA mg^{-1}	5 mV s^{-1}	*ACS Catal.,* 2020, 10, 3945-3957
Pt/C	26.2 mA mg^{-1}	10 mV s^{-1}	*Adv. Funct. Mater.,* 2022, 32, 2110702
Pt/C	24.61 mA mg^{-1}	20 mV s^{-1}	*Angew. Chem. Int. Ed.,* 64, e202418691
Pt/C	48.2 mA mg^{-1}	50 mV s^{-1}	*Joule,* 2019, 3, 2472-2484
Pt/C	9.2 mA mg^{-1}	10 mV s^{-1}	*Electrochimica Acta,* 2020, 341, 136027
Pt/C	14 mA mg^{-1}	10 mV s^{-1}	*ChemElectroChem,* 2016, 3, 605-614
Pt/C	30 mA mg^{-1}	10 mV s^{-1}	*J. Mater. Chem. A,* 2016, 4, 11060-11068
Pt/C	11.5 mA mg^{-1}	50 mV s^{-1}	*J. Electroanal. Chem.,* 2016, 29-36
Pt/C	4.02 mA mg^{-1}	50 mV s^{-1}	*Electrochimica Acta,* 2016, 222, 1455-1463
Pt/C	26.5 mA mg^{-1}	10 mV s^{-1}	*ACS Appl. Mater. Interfaces,* 2017, 9, 27765-27772

Table S6. Comparison DAFC activities of the Pt-DG-1|| Pt-DG-1 with some recently reported Pt-based catalysts.

Catalysts	Pt mass loading (mg cm^{-2})	Pt utilization (mW mg_{Pt}^{-1})	Reference
Pt/C	0.4	18.55	*This work*
Pt-DG-1	0.034	641.18	*This work*
$PtIrZn_2/SiO_2$–CNT–COOH	1	314	Energy Environ. Sci., 2021, 14, 1449
$PtIrZn_2/CeO_2$-ZIF-8	1	91	Energy Environ. Sci., 2021, 14, 1449
PtIr/C	4	18.75	ACS Energy Lett. 2021, 6, 1996
PtIr/C	1	4.17	Int. J. Hydr. Energy. 2014, 39, 5148
Pt-Y_2O_3	0.5	13	J. Cat., 2016, 334, 496
PtAu/C	1	2.64	Appl. Cat. A. 2015, 490, 133
Pt-Ru/C	1.2	4.3	J. Power Sources. 2012, 208, 257
PtRu/C	0.5	6.14	J. Power Sources. 2012, 208, 257
PtIr/C	2	60.5	Joule, 2019, 3, 2472
Pt/C	0.5	9.52	J. Power Sources. 2020, 476, 228454
PtRh/C	2	2.69	Appl. Catal. B-environ., 2015, 174, 136

Table S7. Comparison DAFC activities of the Pt-DG-1||Pt-DG-1 with some recently reported Pt-based catalysts (commercial AEM).

**Catalysts**	**Power density (mW cm^{-2})**	**Pt loading (mg cm^{-2})**	**Reference**
Pt-DG-1	21.8	0.034	*This work*
Pt/C	7.42	0.4	*This work*
$Ag_2Pt_3TiS_6$	8.71	0.45	*Angew. Chem. Int. Ed.* 2025, 64, e202418691
$Pt_{90}Ru_{10}/C$	2	2	*Int. J. Hydr. Energy.* 2017, 42, 193-201
PtIr/C	4.17	1	*Int. J. Hydr. Energy.* 2014, 39, 5148-5152
PtRh/C	5.37	2	*Appl. Catal. B-Environ.* 2015, 174, 136-144
PtAu/C	2.64	1	*Appl. Catal. A-Gen.* 2015, 490, 133-138
Y_2O_3-modified Pt	6.5	0.5	*J. Catal.* 2016, 344, 496-506
Pt-Ru/C	5.16	1.2	*J. Power Sources.* 2012, 208, 257-262
PtRu/C	3.07	0.5	*J. Power Sources.* 2012, 208, 257-262
SnO_2-Pt/C	4.15	0.4	*Electrochim. Acta.* 2015, 173, 364-369
PtC	4.76	0.5	*J. Power Sources.* 2020, 476, 228454

References

[1] a)G. Kresse, J. Hafner, *Phys. Rev. B* **1993**, *47*, 558; b)G. Kresse, J. Hafner, *Phys. Rev. B* **1994**, *49*, 14251; c)G. Kresse, J. Furthmüller, *Comp. Mater. Sci.* **1996**, *6*, 15.

[2] J. P. Perdew, K. Burke, M. Ernzerhof, *Phys. Rev. Lett.* **1996**, *77*, 3865.

[3] P. E. Blöchl, *Phys. Rev. B* **1994**, *50*, 17953.

[4] H. J. Monkhorst, J. D. Pack, *Phys. Rev. B* **1976**, *13*, 5188.

[5] G. Henkelman, A. Arnaldsson, H. Jónsson, *Comp. Mater. Sci.* **2006**, *36*, 354.

[6] J. K. Nørskov, J. Rossmeisl, A. Logadottir, L. Lindqvist, J. R. Kitchin, T. Bligaard, H. Jonsson, *J. Phys. Chem. B* **2004**, *108*, 17886.
